# DYNLT3 overexpression induces apoptosis and inhibits cell growth and migration *via* inhibition of the Wnt pathway and EMT in cervical cancer

**DOI:** 10.3389/fonc.2022.889238

**Published:** 2022-07-29

**Authors:** Jianan Zhang, Qi Shen, Lu Xia, Xueqiong Zhu, Xuejie Zhu

**Affiliations:** ^1^ Center of Uterine Cancer Diagnosis and Therapy Research of Zhejiang Province, Department of Obstetrics and Gynecology, The Second Affiliated Hospital of Wenzhou Medical University, Wenzhou, China; ^2^ Department of Obstetrics and Gynecology, The First Affiliated Hospital of Wenzhou Medical University, Wenzhou, China

**Keywords:** DYNLT3, cervical cancer, proliferation, apoptosis, invasion, migration

## Abstract

The role of the dynein light chain Tctex-type 3 (DYNLT3) protein in the biological behavior of cervical cancer and its relative molecular mechanisms were investigated. Immunohistochemical staining was used to detect DYNLT3 protein expression in cervical cancer tissues. Cell proliferation and apoptosis rates and invasiveness and migratory capacities were determined by CCK-8 assays, BrdU staining assays and colony formation assays, fluorescence activated cell sorting (FACS), wound healing assays, and Transwell invasion assays of cervical cancer cells after DYNLT3 modulation. The expression levels of Wnt signaling pathway- and EMT-related proteins were examined by Western blotting. Furthermore, the effects of DYNLT3 on the tumorigenicity and metastasis of cervical cancer in nude mice were analyzed by performing immunohistochemistry, and we found that the expression level of the DYNLT3 protein was higher in human normal cervical tissues than in cervical cancer tissues. Overexpression of DYNLT3 obviously attenuated the proliferation, migration and invasion of CaSki and SiHa cells, and promoted cell apoptosis. Upregulation of DYNLT3 expression markedly decreased the expression of Wnt signaling pathway-related proteins (Dvl2, Dvl3, p-LRP6, Wnt3a, Wnt5a/b, Naked1, Naked2, β-catenin and C-Myc) and EMT-related proteins (N-cadherin, SOX2, OCT4, vimentin and Snail), and increased the expression of E-cadherin and Axin1. However, the opposite results were observed after down-regulation of DYNLT3 expression. Up-regulation of DYNLT3 expression significantly inhibited tumor growth in a nude mouse model, while downregulation of DYNLT3 showed the opposite results. In addition, the major metastatic site of cervical cancer cells in mice was the lung, and downregulation of DYNLT3 expression increased cancer metastasis *in vivo*. DYNLT3 exerted inhibitory effects on cervical cancer by inhibiting cell proliferation, migration and invasion, promoting cell apoptosis *in vitro*, and inhibiting tumor growth and metastasis *in vivo*, possibly by suppressing the Wnt signaling pathway and the EMT.

## Introduction

Cervical cancer is the fourth most commonly diagnosed cancer and the fourth leading cause of cancer mortality among females worldwide, accounting for nearly 6.5% of the total new cancer cases in women and 7.7% of cancer mortalities (1). Cervical cancer is a complex and multifactorial disease, mostly due to persistent infection with human papillomavirus (HPV). In addition, chemical or microbial cofactors or immune or sex hormones can participate in the initiation of cervical (pre)neoplastic lesions (2). Cervical cancer therapies include chemotherapy (3). Because advanced inoperable cervical cancer is a challenge to treat, the development of targeted therapies to improve the therapeutic efficacy has become a research hotspot.

Dynein light chain Tctex-type 3 (DYNLT3), as a component of the cytoplasmic dynein complex, is distributed in different locations, such as the cytoplasm, the centromere, nucleus, and microtubules (4). DYNLT3 is involved in linking dynein to cargos and to govern its function and binds with the mitotic protein BUb3 to control mitosis and meiosis progression (5). To date, reports of the biofunctions of DYNLT3 in malignant tumors have been inconsistent. In esophageal squamous cell carcinoma, DYNLT3 expression was significantly decreased and may serve as a tumor suppressive factor (6). However, in salivary gland adenoid cystic carcinoma, DYNLT3 was predicted to be a candidate oncogene, and in our previous ovarian cancer research, DYNLT3 also showed tumor-promoting effects by facilitating cell proliferation and invasion in ovarian cancer cells (7, 8). Herein, we aimed to explore whether DYNLT3 is linked to the occurrence and development of cervical tumorigenesis.

In this study, DYNLT3 expression was measured by immunohistochemical (IHC) staining in cervical tumor specimens. Subsequently, lentiviruses were transfected to up- or downregulate the expression of DYNLT3 in cell lines and in nude mouse models. The proliferation, apoptosis, and motility of cancer cells were investigated by CCK-8 assay, BrdU staining assay, FACS, wound healing assay, and Transwell invasion assays, respectively. Nude mice, *in vivo* imaging and small-animal technology were used to analyze the effects of DYNLT3 on the tumorigenicity and metastasis of cervical cancer. The expression of Wnt pathway and epithelial-mesenchymal transition (EMT) markers was examined by Western blotting. Our study investigated the functions of DYNLT3 and its underlying molecular mechanism in cervical cancer.

## Results

### Expression of the DYNLT3 protein is decreased in cervical tumor tissues

**Figure 1:**
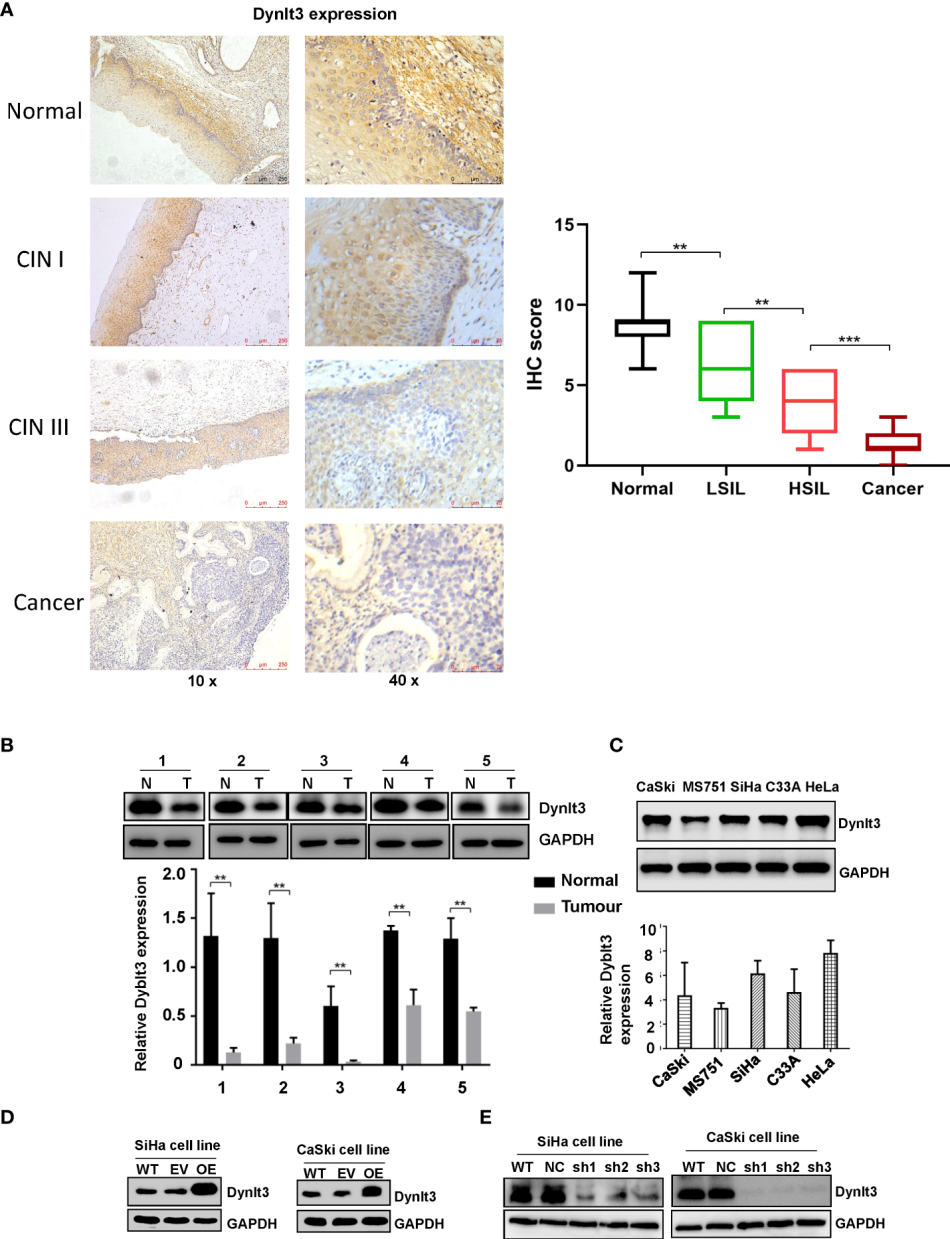
Expression of the DYNLT3 protein in cervical cancer tissues and cell lines. **(A)** Left panel: The expression of the DYNLT3 protein in normal cervical tissues, CIN1, CINII and cervical cancer tissues was detected by immunohistochemistry. Right panel: IHC scores were shown in normal tissues, LSIL, HSIL and cancer. **(B)** Top panel: The expression of the DYNLT3 protein in cervical cancer cell tissues was measured by Western blotting. N, normal tissues; T, tumor tissues. Bottom panel: The quantitative data are illustrated for Dynlt3 expression. ** *P* < 0.01 vs. normal tissues. **(C)** Top panel: The expression of the DYNLT3 protein in cervical cancer cell lines was measured by Western blotting. Bottom panel: Quantitative data are presented for Dynlt3 expression. **(D)** Western blotting data showed that upregulation of DYNLT3 was mediated by lentivirus transfection in cervical cancer cells. WT, wild type; EV, empty vector; OE, overexpression of DYNLT3. **(E)** Downregulation of DYNLT3 is shown in cervical cancer cells. WT, wild type; NC, negative control; sh, DYNLT3 shRNA. ***P < 0.001.

The expression level of the DYNLT3 protein was highest in human normal cervical tissues among CIN1, CINII and cervical cancer tissues as determined by immunohistochemistry (**Figure 1A**). In line with this finding, Western blotting data demonstrated that higher expression of DYNLT3 was observed in normal cervical tissues (**Figure 1B**). Moreover, CaSki, MS751, SiHa, C-33A and HeLa cell lines were selected to detect the expression of DYNLT3 protein by Western blotting analysis. Our results demonstrated that DYNLT3 was expressed in the five cervical cancer cell lines (**Figure 1C**).

### DYNLT3 inhibits the proliferation of cervical cancer cells

**Figure 2:**
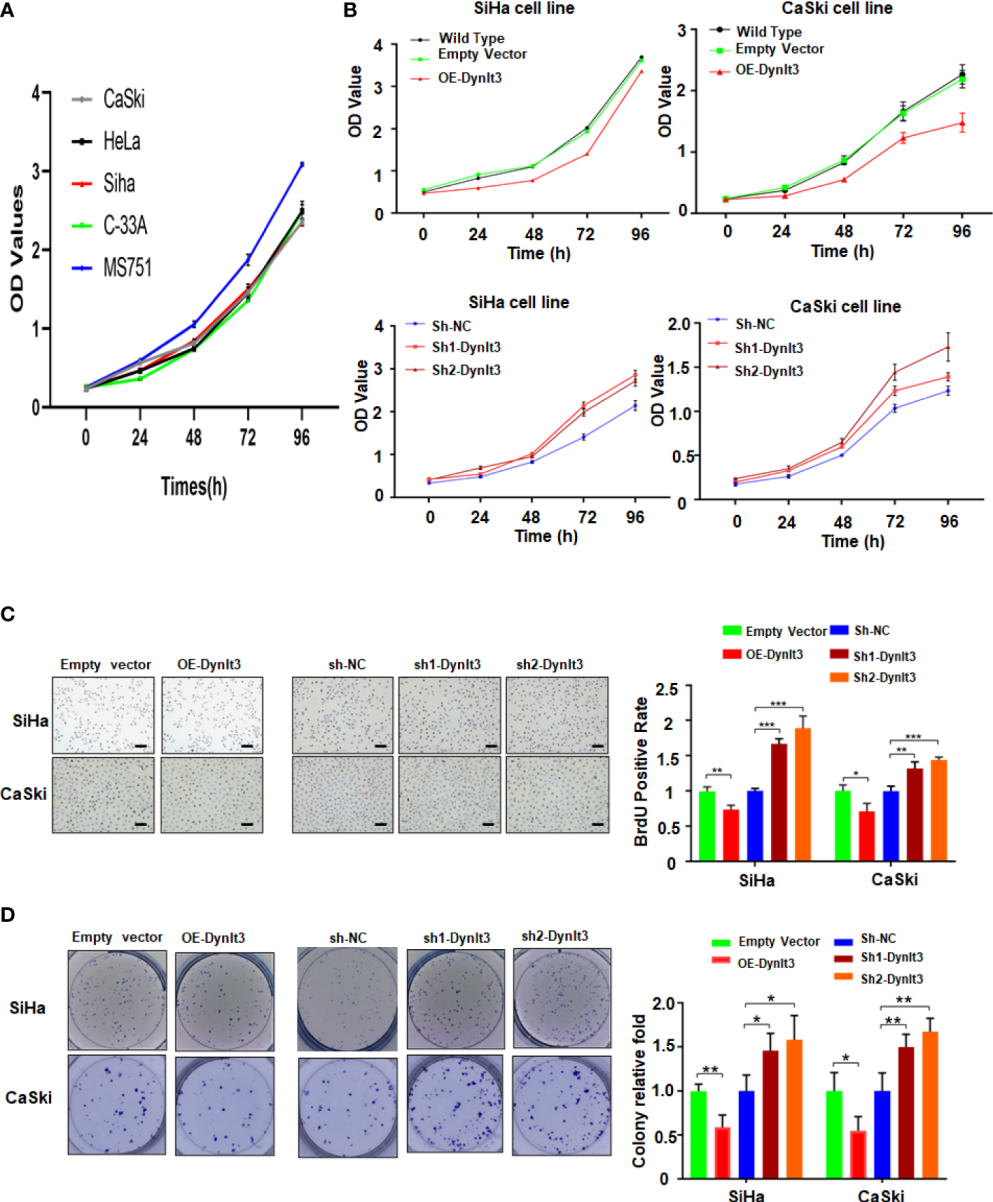
Effects of DYNLT3 on the proliferation of cervical cancer cells. **(A)**: The viability of several cervical cancer cell lines was measured by CCK-8 assay. **(B)**: The effects of DYNLT3 modulation on the viability of cervical cancer cells were examined by CCK-8 assay. **(C)**: Left panel: The effects of DYNLT3 on the proliferation of cervical cancer cells were tested by BrdU staining assay. Right panel: The quantitative data are illustrated for the left panel. Scale bar: 100 μM. * *P*< 0.05, ** *P* < 0.01, *** *P* < 0.001. **(D)**: Left panel: The effects of DYNLT3 on the proliferation of cervical cancer cells were measured by colony formation assay. Right panel: The quantitative data are illustrated for the left panel. * *P*< 0.05, ** *P* < 0.01.

In CaSki and SiHa cells, DYNLT3 protein expression was distinctly upregulated when the cells were transfected with lentivirus overexpressing of DYNLT3, while these cells transfected with lentivirus expressing shRNA-DYNLT3 exhibited dramatic downregulation of DYNLT3 expression, and the control vector showed no changes in DYNLT3 expression (**Figure 1D**). Then, sh1 and sh2 shRNA-DYNLT3 were selected for subsequent experiments according to the protein inhibition efficacies in SiHa and CaSki cells (**Figure 1E**). Three experimental techniques (CCK-8, BrdU staining and colony formation assays) were used to analyze cell proliferation. The viability of several cervical cancer cell lines has a similar trend (**Figure 2A**). Moreover, our results consistently demonstrated that cell proliferation in the DYNLT3-overexpressing group was obviously inhibited in both CaSki and SiHa cell lines (*P*<0.05, **Figures 2B–D**). Conversely, cell proliferation in both cell lines with DYNLT3 knockdown was enhanced compared with the corresponding control group (*P*<0.05, **Figures 2B–D**).

### DYNLT3 promotes the apoptosis of cervical cancer cells

**Figure 3:**
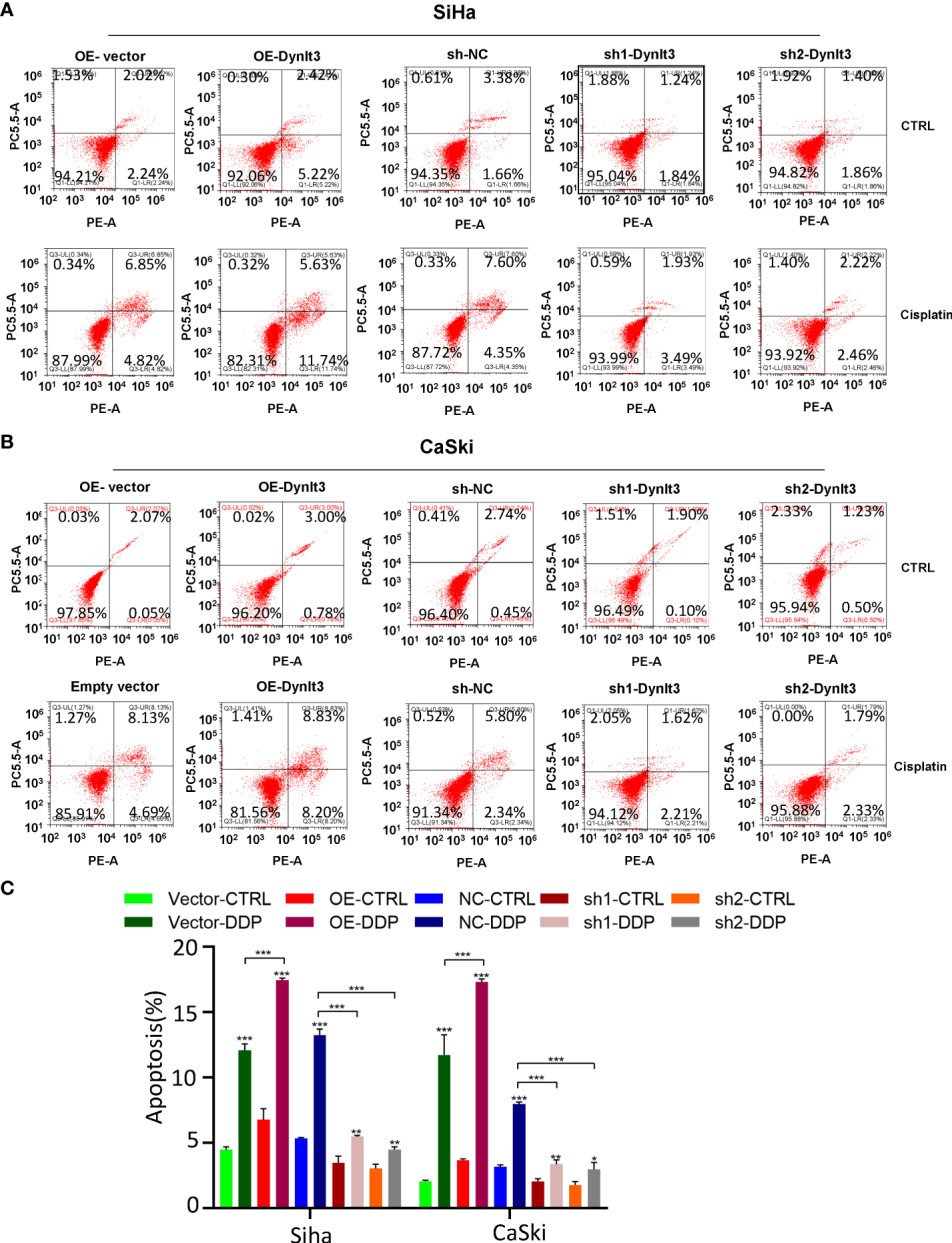
Effects of DYNLT3 on the apoptosis of cervical cancer cells. **(A)**: The effects of DYNLT3 overexpression or knockdown on the apoptosis of SiHa cervical cancer cells after 4 nM cisplatin treatment for 24 hours were measured by flow cytometric analysis. **(B)**: The effects of DYNLT3 overexpression or knockdown on the apoptosis of CaSki cervical cancer cells after 4 nM cisplatin treatment for 24 hours were detected by flow cytometry. **(C)**: The quantitative data for cell apoptosis. * *P*< 0.05, ** *P* < 0.01, *** *P* < 0.001.

**Figure 4:**
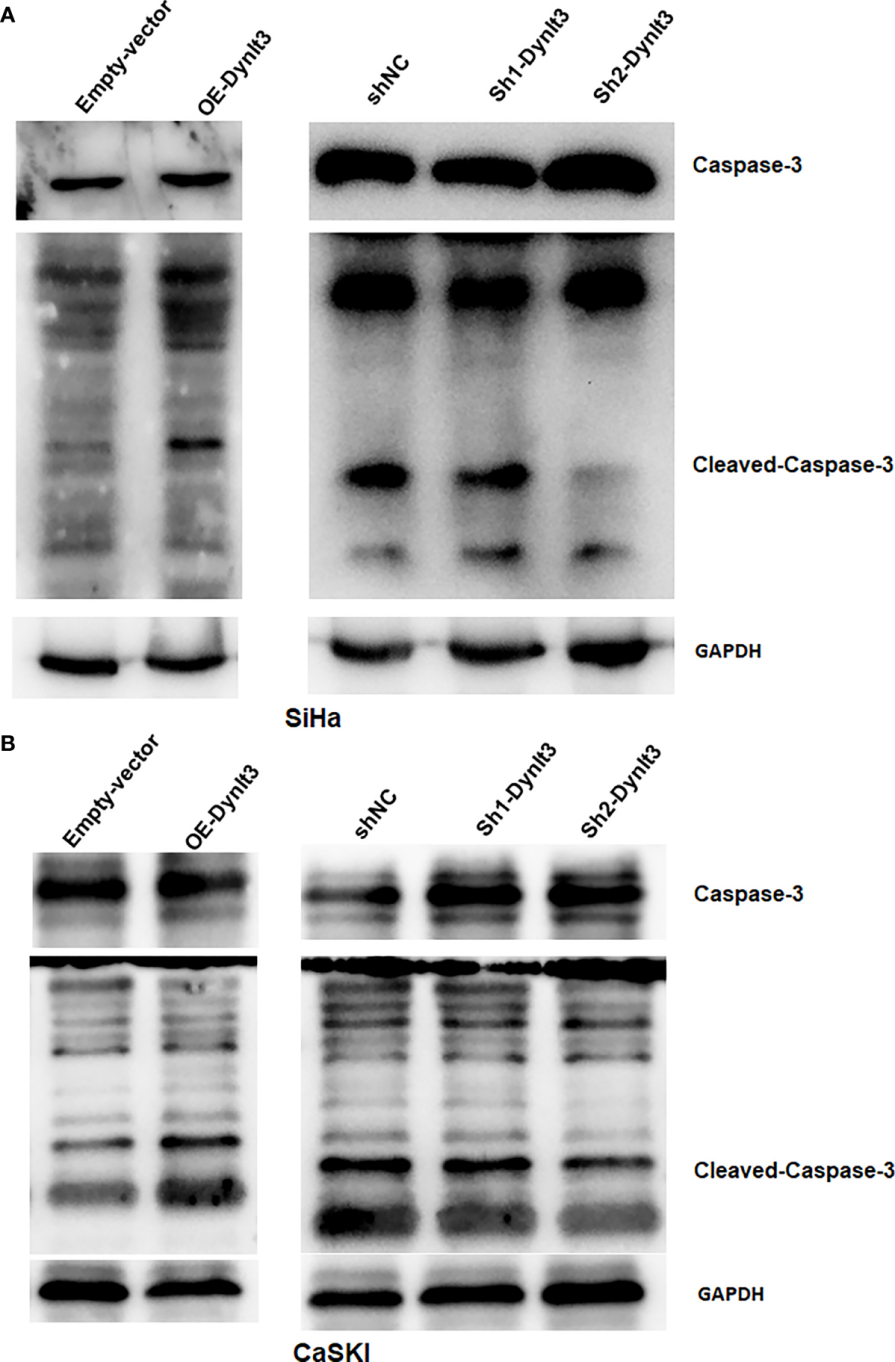
Effects of DYNLT3 on the caspase-3 expression in cervical cancer cells. **(A, B)**: Western blotting was performed to measure the expression of caspase-3 and cleaved caspase-3 in SiHa cells **(A)** and CaSki cells **(B)** after DYNLT3 modulation.

In CaSki and SiHa cell lines, data from flow cytometry assays demonstrated that the percentage of apoptotic dead cells was markedly elevated in the DYNLT3-overexpressing group compared with that in the control vector group (**Figures 3A,B**). Conversely, the percentages of apoptotic dead cells were significantly decreased in both DYNLT3 knockdown groups compared with the corresponding control group (*P*<0.05, **Figures 3A–C**). Moreover, DYNLT3 overexpression also enhanced cisplatin-induced apoptosis in both cell lines, while DYNLT3 knockdown inhibited cisplatin-mediated apoptosis (**Figures 3A–C**). Furthermore, we observed that DYNLT3 can regulate the expression of cleaved caspase 3 in SiHa and CaSki cells, which could contribute to cell apoptosis (**Figures 4A,B**). Notably,

### DYNLT3 inhibits the migration and invasion of cervical cancer cells

**Figure 5:**
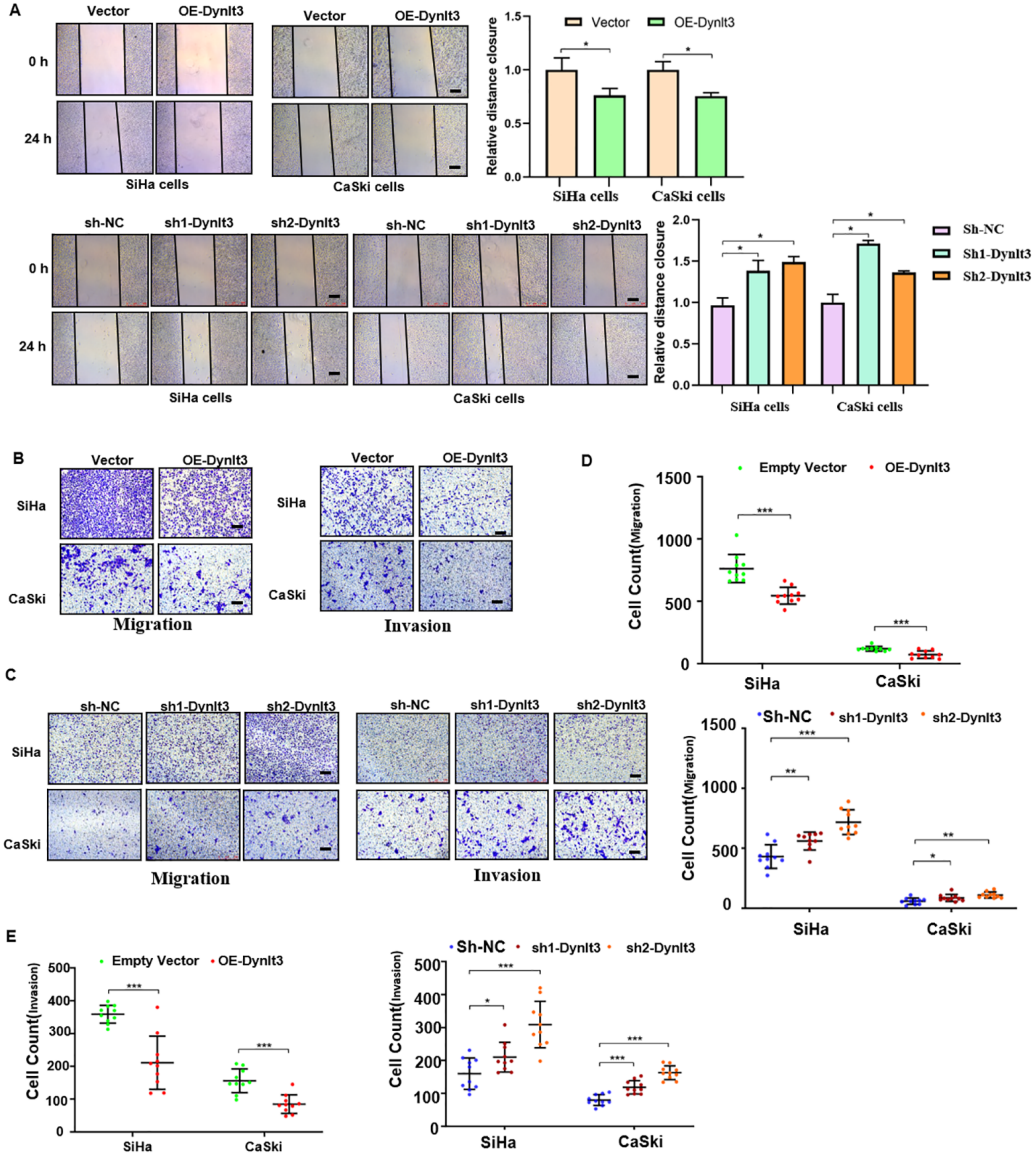
Effects of DYNLT3 on the migration and invasion of cervical cancer cells. **(A)** Left panel: The effects of DYNLT3 on the migration of cervical cancer cells were detected by wound healing assay. Right panel: The quantification of wound closure is shown. **(B)** The effects of DYNLT3 overexpression on the migration and invasion of cervical cancer cells were measured by Transwell assay. **(C)** The effects of DYNLT3 knockdown on the migration and invasion of cervical cancer cells were detected by Transwell assay. Scale bar: 250 μM. **(D)** The quantification of cell migration is illustrated. *P < 0.05, **P < 0.01, ***P < 0.001. **(E)** The quantification of cell invasion is presented. *P < 0.05, **P < 0.01, ***P < 0.001.

Wound healing assays and Transwell migration assays were used to assess the migration abilities of CaSki and SiHa cells. The results revealed that overexpression of DYNLT3 markedly reduced the migration ability compared with the control vector group (**Figures 5A–C**). Conversely, the migration ability of cells was significantly increased in both DYNLT3 knockdown groups (**Figures 5A–C**). Transwell invasion assays were used to assess the invasion abilities of CaSki and SiHa cells, and the results showed that overexpression of DYNLT3 significantly reduced the number of cells that invaded the membrane. Conversely, cells in both DYNLT3 knockdown groups exhibited increased invasion abilities (**Figures 5B–E**).

### DYNLT3 affects the Wnt signaling pathway in cervical cancer cells

**Figure 6:**
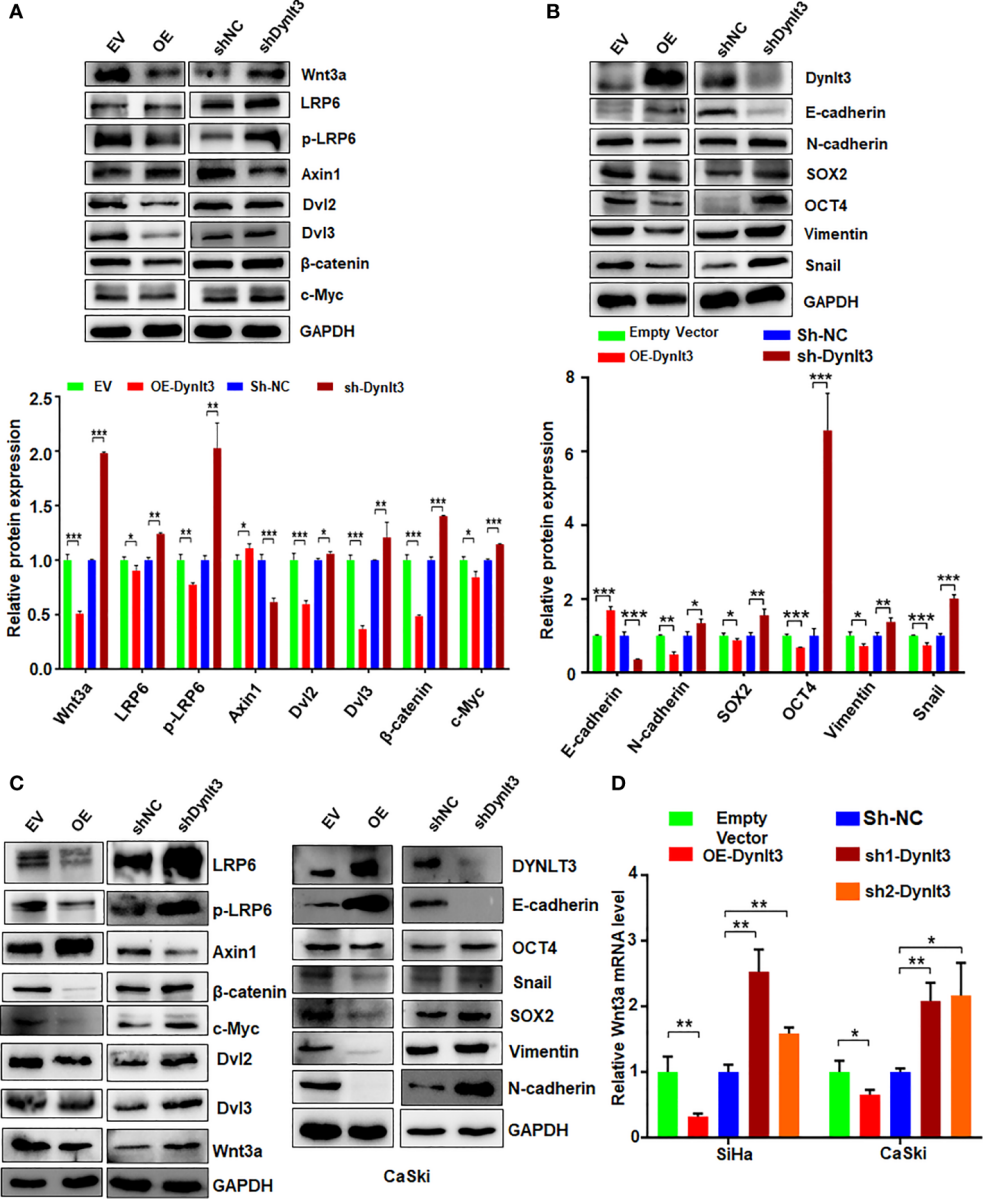
Effects of DYNLT3 on the expression of Wnt signaling pathways. **(A)**: Top panel: The effects of DYNLT3 on the expression of the Wnt pathway-related proteins were explored by Western blotting in SiHa cervical cancer cells. Bottom panel: Quantification of the Wnt pathway. * *P*< 0.05, ** *P* < 0.01, *** *P* < 0.001. sh-Dynlt3: sh2-Dynlt3. **(B)**: Top panel: The effects of DYNLT3 on the expression of the EMT markers were measured by Western blotting in SiHa cervical cancer cells. Bottom panel: Quantification of the Wnt pathway. * *P*< 0.05, ** *P* < 0.01, *** *P* < 0.001. **(C)**: The effects of DYNLT3 on the expression of the Wnt pathway-related proteins were explored by Western blotting in CaSki cervical cancer cells. **(D)**: The mRNA levels of Wnt3a were measured by real-time RT–PCR in SiHa and CaSki cells after Dynlt3 modulation. * *P*< 0.05, ** *P* < 0.01, *** *P* < 0.001.

To explore the molecular mechanism of DYNLT3-mediated tumorigenesis, we measured the expression levels of several cellular signaling pathways. Among these signaling pathways, Wnt signaling was significantly regulated by DYNLT3. As shown in **Figures 6A–C**, when DYNLT3 expression was upregulated, the expression levels of Wnt signaling pathway-related proteins (Dvl2, Dvl3, p-LRP6, LRP6, Wnt3a, Wnt5a/b, Naked1, Naked2, β-catenin and C-Myc) were decreased, while the Axin1 expression was increased. In contrast, the expression levels of these above proteins were reversed when DYNLT3 was downregulated (**Figures 6A–C**).

### DYNLT3 affects the expression of the EMT markers in cervical cancer cells

**Figure 7:**
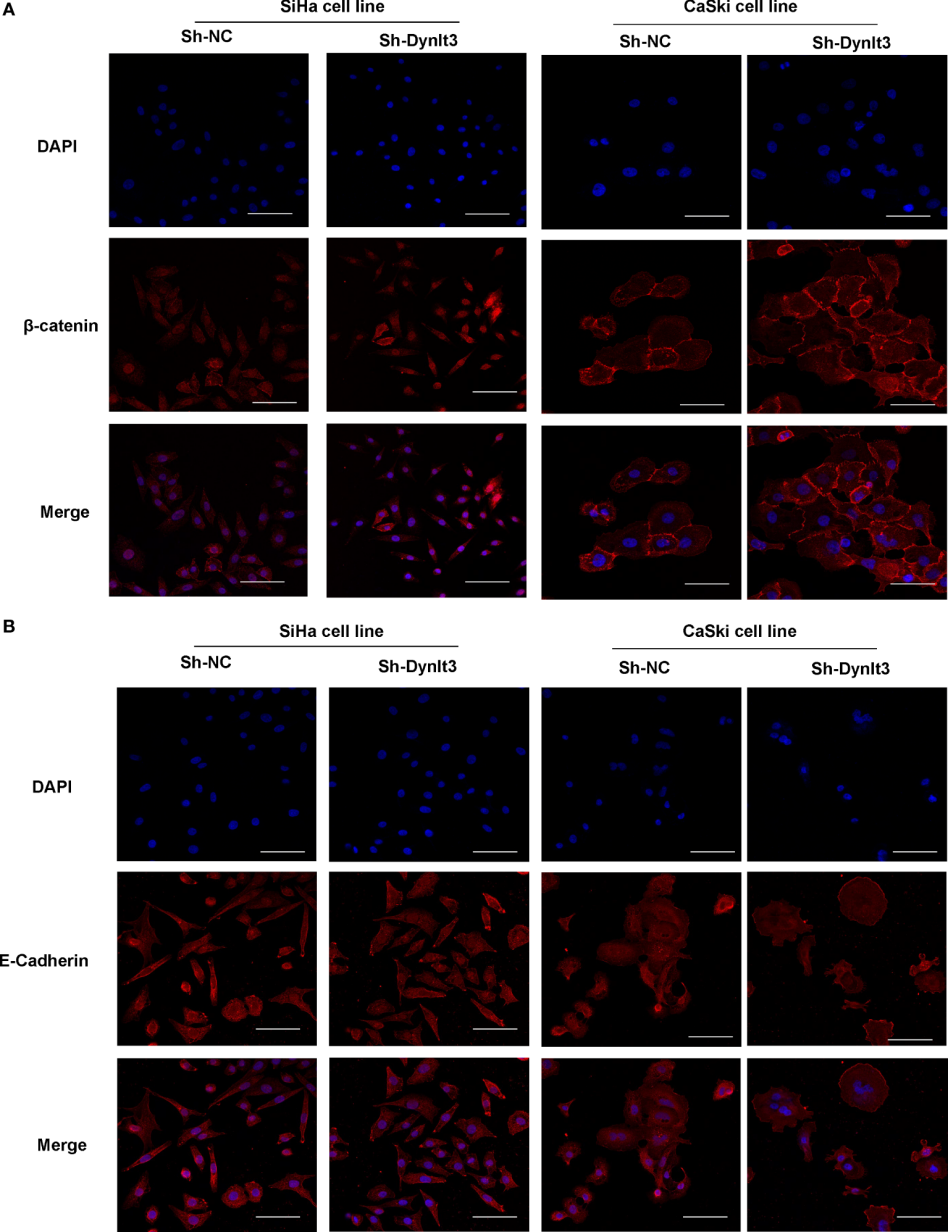
Effects of DYNLT3 on the β-catenin and E-cadherin in cervical cancer cells. **(A)**: Immunofluorescence was conducted to detect the location of β-catenin in cervical cancer cells after DYNLT3 knockdown. **(B)**: Immunofluorescence was performed to detect the expression of E-cadherin in cervical cancer cells after DYNLT3 knockdown.

Western blotting assay data showed that upregulation of DYNLT3 suppressed the expression of EMT-related proteins (N-cadherin, SOX2, OCT4, vimentin and Snail) and elevated the E-cadherin expression (**Figures 6B, C**). The opposite results were shown in DYNLT3-knockdown group, indicating that DYNLT3 regulates EMT marker expression (**Figures 6B, C**). Our RT–PCR data also showed that DYNLT3 regulated the mRNA levels of Wnt3a in both cervical cancer cell lines (**Figure 6D**). Moreover, we conducted immunofluorescence and observed that DYNLT3 knockdown led to a decreased E-cadherin at the membrane and increased nuclear β-catenin (**Figures 7A, B**).

### The effects of DYNLT3 on the tumor growth and metastasis in a mouse model

**Figure 8:**
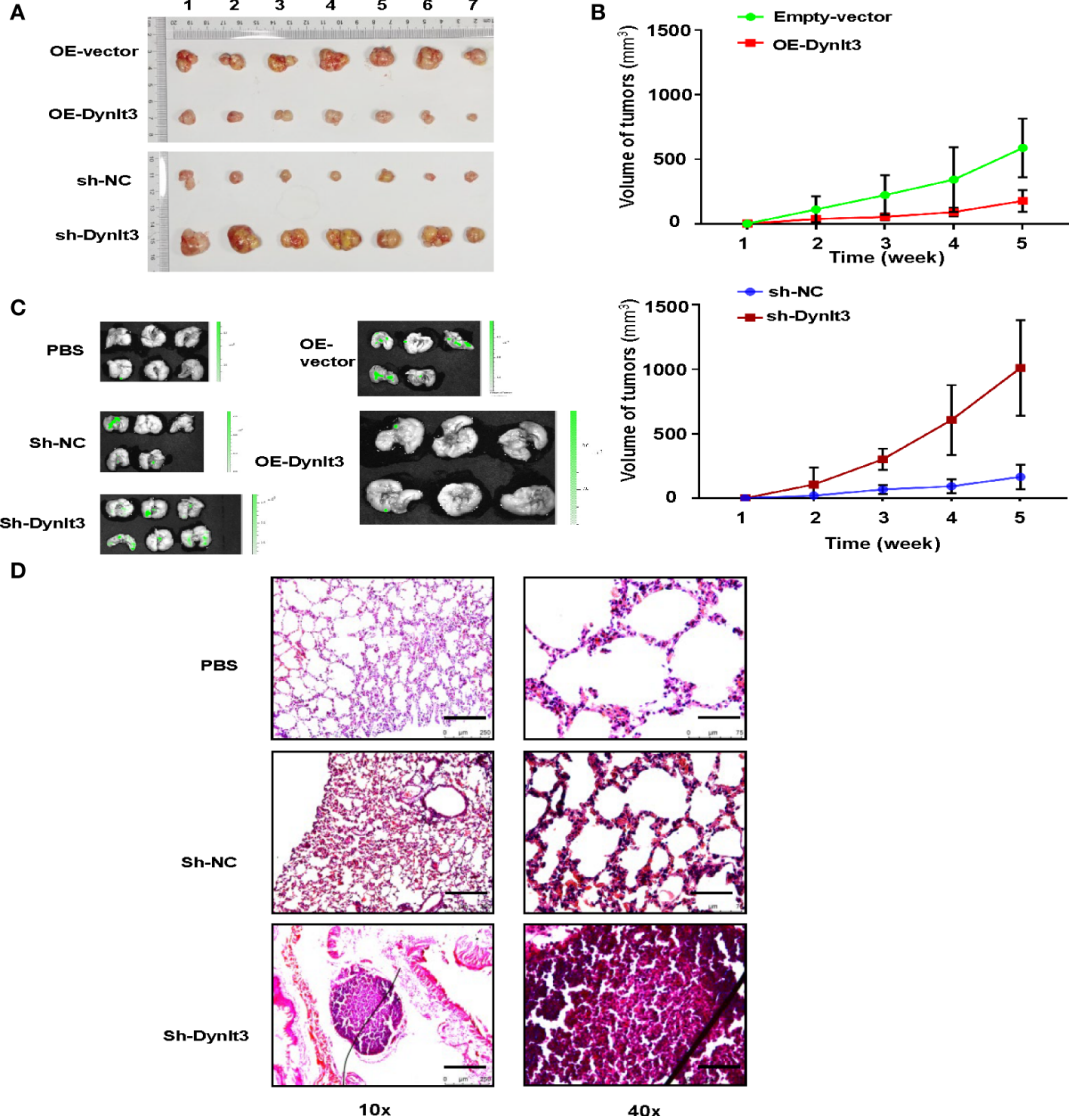
Effects of DYNLT3 on the tumor growth and metastasis in a mouse model. **(A)**: DYNLT3 inhibited the tumor growth in nude mice bearing human SiHa cervical cancer cells. Tumor sizes are shown. Numbers: tumor sample numbers. **(B)**: Tumor volumes are presented in mice bearing SiHa cells after DYNLT3 modulation. **(C)**: Downregulation of DYNLT3 promoted the lung metastasis in mice bearing human SiHa cervical cancer cells. **(D)**: HE staining was performed in lung tissues from nude mice bearing SiHa cells after DYNLT3 modulation.

A nude mouse model bearing human cervical cancer cells was used to evaluate the effects of DYNLT3 on the tumor growth *in vivo*. Tumor volume was detected every week. Compared with the control group, upregulation of DYNLT3 expression significantly inhibited tumor growth, while downregulation of DYNLT3 showed the opposite results (*P*< 0.05, **Figures 8A, B**). In addition, *in vivo* imaging of small animal’s technology was used to analyze the metastasis sites and quantity of cervical cancer cells (green fluorescence). As shown in **Figure 8C**, the major metastatic site of cancer cells was the lung, and the green fluorescence quantity of lung cancer was increased when the expression of DYNLT3 was downregulated. Moreover, downregulation of DYNLT3 increased the cancer metastasis in the lung (**Figure 8D**).

## Discussion

In this study, we reported that the expression level of DYNLT3 was lower in the human cervical cancer tissues than that in normal cervical tissues. In addition, upregulation of DYNLT3 attenuated proliferation, reduced migration and invasion, and enhanced cell apoptosis in cervical cancer cells. In addition, upregulation of DYNLT3 inhibited the tumor growth and metastasis in nude mice bearing human cervical cancer cells. Conversely, the opposite results were observed when DYNLT3 expression was downregulated. Furthermore, the Wnt signaling pathway and EMT-related proteins were involved in the molecular mechanism of DYNLT3-mediated antitumor activity in cervical cancer. Altogether, these results indicate that DYNLT3 may have a crucial function in the occurrence and development of cervical cancer, which may provide a new approach for combating cervical cancer.

DYNLT3 is a member of the dynein light chain Tctex family, which mainly localizes in the microtubules, the cytoplasm and the nucleus, and is related to the occurrence and development of many tumors (9). Through a whole-genome screen assay of salivary adenoid cystic carcinoma, DYNLT3 is discovered to be an oncogene (7). We previously found that the expression of DYNLT3 protein was low in normal ovarian epithelium and was increased in serous cystadenoma and serous cystadenocarcinoma. Interestingly, there is a discrepancy of DYNLT3 expression assessed by IHC and western blotting in cervical cancer tissues and cell lines. We believe that the expression of DYNLT3 in cancer tissues is more important than that in cell lines. Moreover, overexpression of DYNLT3 promoted cell proliferation and enhanced invasiveness and migratory ability, and overexpression of DYNLT3 was associated with worse prognosis, indicating that DYNLT3 may participate in ovarian carcinogenesis and progression (8). However, the expression of DYNLT3 was downregulated in esophageal squamous cell carcinoma and may play a role in tumor suppression (6). In the present study, DYNLT3 repressed proliferation and motility and stimulated cell apoptosis in cervical cancer. These results showed that the functions of DYNLT3 in different cancers are inconsistent, indicating that the role of DYNLT3 may be organ-dependent.

EMT is a reversible biological process that can induce an epithelial cell phenotype to obtain mesenchymal phenotypes and promote tumor migration and invasion (10, 11). Recent studies have shown that the EMT exerts an important influence on the progression of cervical cancer (12, 13). Specific components of the main EMT regulators, such as E-cadherin, N-cadherin, vimentin, Snail, SOX2, and OCT4, are known to play roles in EMT progression (14–17). E-cadherin, as a tumor suppressor, could inhibit the peripheral infiltration and migration of tumor cells, which is decreased in the EMT process and cervical cancer development (18). However, N-cadherin is related to embryonic differentiation and formation, and is increased during the EMT process and cervical cancer progression (19–21). Vimentin, as an important cytokine that stimulates tumor progression, is mainly distributed in mesenchymal tissues and is increased during the EMT process and cervical cancer progression (22). Snail, as a member of the zinc finger family of transcription factors, can bind to the E-cadherin gene to induce vimentin expression, and the high expression of Snail is closely related to the EMT process in cervical cancer (23). Our results revealed that DYNLT3 is involved in EMT *via* suppression of N-cadherin, vimentin, Snail, SOX2, and OCT4, and upregulation of E-cadherin expression.

E-cadherin, N-cadherin and vimentin have been implicated in the Wnt signaling pathway (24, 25). Because DYNLT3 regulated these EMT regulators, we explored whether DYNLT3 can regulate the Wnt signaling pathway. The Wnt signaling pathway, which consists of multiple factors (β-catenin, Dvl2, LRP6, Wnt, Naked, C-Myc and Axin1, etc.) involved in cell proliferation, differentiation, migration, and polarity, is associated with many cancers, including cervical cancer (26–28). β-catenin, a pivotal component of the Wnt signaling pathway, is essential for development and carcinogenesis (29). β-catenin plays an oncogenic role and could be a therapeutic target in cervical cancer (30, 31). Dvl2, as a key mediator of the Wnt pathway, participates in the progression of several cancers. Suppression of Dvl2 increased cisplatin sensitivity *via* inhibition of the Wnt/β-catenin pathway in lung cancer cells, and blocking Dvl2/Snail signaling suppressed metastasis and reversed chemoresistance in colorectal cancer cells (32, 33). However, there is no report on the relationship between Dvl2 and cervical cancer. In the present study, our results showed that DYNLT3 could inhibit Dvl2 expression in the Wnt pathway in cervical cancer. Dvl3, as a multivalent scaffold with several well-known domains, is significantly upregulated in cervical cancer and participates in cervical cancer oncogenesis *via* Wnt/β-catenin activation (34). LRP6, a Wnt coreceptor, shows increased expression in cervical cancer and may serve as an oncoprotein by blocking Wnt/β-catenin signaling (35). C-Myc acts as an oncogene to control multiple biological processes, and its high expression could promote the proliferation and metastasis of cervical cancer, which may play a synergistic role in the pathogenesis of cervical cancer (36, 37). The tumor suppressor AXIN1, acting as a negative upstream regulator of β-catenin levels and localization, inhibits the expression of Wnt and β-catenin target genes, which represents a potential therapeutic strategy in cervical cancer (38). Our results indicate that DYNLT3 inhibits the Wnt pathway by decreasing the expression of Dvl2, Dvl3, p-LRP6, Wnt3a, Wnt5a/b, Naked1, Naked2, β-catenin and C-Myc, and enhancing Axin1 expression. Characterizing the mechanisms by which DYNLT3 regulates the Wnt pathway and EMT process could improve our understanding of the tumorigenesis and may allow for the development of improved therapeutics for cervical cancer.

## Conclusion

In conclusion, DYNLT3 plays a potential role in cervical cancer and serves as a tumor suppressor because DYNLT3 overexpression inhibited cancer cell proliferation, migration and invasion, and promoted apoptosis, and inhibited the tumor growth and metastasis *in vivo*, possibly by regulating the Wnt signaling pathway and EMT process. It is important to note that our study does not provide the molecular mechanisms by which DYNLT3 regulates WNT pathway in cervical cancer cells. Advances in understanding over the mechanism of DYNLT3 in cervical cancer may benefit for the development of therapeutic strategies and novel treatment agents.

## Materials and methods

### Tissue specimens

From October 2017 to December 2019, 20 cervical cancer tissues from cervical cancer patients who underwent radical surgery were selected, and 20 normal cervical specimens from patients with uterine fibroids who underwent total hysterectomy during the same period at the Second Affiliated Hospital of Wenzhou Medical University were selected as normal controls. All diagnoses were confirmed surgically and pathologically. Written informed consent was obtained from all patients. This study was approved by the ethics committee of the Second Affiliated Hospital of Wenzhou Medical University (KY-2017-100).

### Immunohistochemistry

In the IHC assay, 4-μm tissue was cut from paraffin-embedded blocks, adhered and dried on slides. Slides were used for IHC analysis followed by: 65°C for 1 h, dimethylbenzene(I) for 20 min, dimethylbenzene(II) for 20 min, 100% ethanol(I) for 10 min, 100% ethanol(II) for 10 min, 95% ethanol(I) for 10 min, 95% ethanol(II) for 10 min, 85% ethanol for 10 min, 75% ethanol for 10 min, and water for 5 min. Next, the slides were retrieved by citric acid buffer (PH 6.0) (C1010-2L, Solarbio, China) microwave antigen retrieval. After incubating in 3% H_2_O_2_ for 20 min, the slides were washed three times with PBS buffer (P1010-2L, Solarbio, China) and blocked with 5% goat serum (SL038, Solarbio, China) for 30 min at 37°C. The expression of DYNLT3 was measured by IHC using an anti-DYNLT3 antibody (ab121209, 1:100; Abcam, UK) at 4°C for overnight. IgG HRP and DAB staining protocols were performed according to the specification of the Rabbit Tow-step kit (PV-6001, ZSGB-BIO, CHN). The staining areas and cumulative light density values of the images were analyzed using Image-Pro Plus (IPP) software.

### Production of lentivirus and transfection of target cells

Human DYNLT3 cDNA was inserted into the PLVX-IRES-ZsGreen1 vector. Small hairpin RNAs (shRNAs) targeting DYNLT3 with sequences (sh1-CCG GTC TAT ACA GCA TCG TTT AAA TCT CGA GAT TTA AAC GAT GCT GTA TAG ATT TTT G-, sh2-CCG GTG ATG GAA CCT GTA CCG TAC TCG AGT ACG GTA CA G GTT CCA TCT TTT TG-, sh3-CCG GGC AAT ATT CTT GTA GGA ATC TCG AGA TTC CTA CAA GAA TAT TGC TTT TTG-) were separately inserted into the TRC2-pLKO-puro-shC vector. These vectors were cotransfected with two packing plasmids, psPAX2 and pMD2.G (Addgene, Inc.), into the HEK293T cells by Lipofectamine 2000 (Thermo Fisher Scientific, USA) to produce lentiviral particles, and those particles were then harvested to infect cervical cancer cell lines. Green fluorescent protein-positive cells were used with flow cytometry to select cells with stable overexpression of DYNLT3 cells, and downregulation of DYNLT3 was selected by 2 μg/ml puromycin.

### Cell counting kit-8 (CCK-8) assay

Cervical cancer cell lines (CaSki, MS751, SiHa, C-33A, and HeLa cell lines) were cultured in RPMI-1640 or DMEM medium with 10% fetal bovine serum (FBS) and 1% penicillin–streptomycin maintained in culture incubators with 5% CO_2_ at 37°C. All cell lines were purchased from the National Collection of Authenticated Cell Cultures (Shanghai, China). A total of 3000 cells were plated in each well with 100 ul of medium to analyze the viability of cervical cancer cells. The CCK-8 assay was utilized as described for Cell Counting Kit-8 (CK04, Dojindo, Japan) every time, and an OD value of 0 h was used as the initial value. All the experiments were repeated at least 3 times.

### Bromodeoxyuridine (BrdU) staining and colony formation assays

Cells were incubated in 6-well plates, and a BrdU staining assay was conducted as described before (39). After cells were cultured overnight, and BrdU was added for 8 h in culture incubators with 5% CO_2_ at 37°C. Then, cells were fixed with 4% paraformaldehyde at room temperature for 30 min, followed by incubation with 0.2% Triton X-100 for 10 min. The cells were incubated with 3% BSA for 1 h and then with anti-BrdU antibody (CST, 1:200, Cat. No. 5292S) at 4°C overnight. The cells were incubated with the secondary antibody for 90 min, stained with DAB for 5 min and counterstained with hematoxylin for 3 min. Positive cells had the brown nuclei, suggesting that those cells were in the proliferation phase. Cells were incubated in 6-well plates (1000 cells/well) for 1-3 weeks to allow visible colony formation. The colonies were fixed with 4% paraformaldehyde for 15 min, and then stained with crystal violet for 15 min. Then, the colonies were washed using PBS and finally counted under a microscope.

### Flow cytometric assay of apoptosis

Cells were incubated in 6-well plates. Then, the cells were washed with PBS, resuspended in binding buffer and incubated with Annexin V-PE and 7-AAD for 15 min at 4°C in the dark. Flow cytometry was utilized for apoptosis analysis as described previously (40).

### Western blotting analysis

The proteins were obtained by lysis buffer (50 mM Tris-HCl pH 7.4, 150 mM NaCl and 0.5% NP-40), and the protein concentration was detected by BCA protein assay. The proteins were separated by SDS–PAGE and transferred onto PVDF membranes as described previously (40). The primary anti-DYNLT3 antibody (1:500) was obtained from Abcam Biotechnology Company. The antibodies against GAPDH (1:3000), c-Myc (5605, 1:1000, CST), Wnt2a (2721, 1:2000, CST), phospho-LRP6 (2568, 1:1000, CST), LRP6 (3395, 1:1000, CST), Dvl2 (3224, 1:1000, CST), Dvl3 (3218, 1:1000, CST), Axin1 (2087, 1:1000, CST), β-Catenin (8480, 1:3000, CST), SOX2 (3579, 1:1000, CST), E-Cadherin (14472, 1:2000, CST), N-Cadherin (13116, 1:2000, CST), OCT4 (2750, 1:1000, CST), Vimentin (5741, 1:2000, CST), Snail (3879, 1:1000, CST) were purchased from CST Biotechnology Company (Danvers, MA, USA). The membranes were incubated with primary antibodies overnight in a cold room after the membranes were blocked with 5% milk. The membranes were treated with an ECL kit and visualized by a GenoSens 2000 Touch machine. AlphaEaseFC 4.0 software was used to quantify the protein bands.

### Wound healing assay

Cells were incubated in 6-well plates and allowed to grow until they were more than 90% confluence. A pipette tip was used to make a wound on the cell layer. The cells were maintained in an incubator for 24 hours. ImageJ software (NIH, MD, USA) was utilized to assess the wound area. The percentages of the wound areas were estimated at 24 h compared with the area at 0 h in each group.

### Transwell assays for migration and invasion

In the migration assay, the upper inserts were seeded in triplicate at 20,000 cells/well for shRNA transfection and 10,000 cells/well for vector transfection in medium without serum. Two hundred microliters of medium with 10% FBS was added to each lower chamber. After 24 h, cells that did not penetrate into the membrane were wiped with a cotton swab. Then, 4% paraformaldehyde was used to fix invaded cells for 20 min. Finally, the invaded cells were counted by a microscope. In the invasion assay, Matrigel was coated on the chamber bottom, and the other steps were the same as above.

### Real‐time quantitative PCR analysis

Total RNA was extracted from cells using RNA-easy Isolation Reagent (R701-01, Vazyme) following the manufacturer’s instructions. Reverse transcription was performed using TransScript All-in-One First-Strand cDNA Synthesis SuperMix for qPCR (P20607, TransGen Biotech, China), and the reaction was blended with PerfectStart Green qPCR SuperMix (P20604, TransGen Biotech, China) and run on a BIO-RAD CFX96 Real-time PCR system as follows: 94 °C 30 sec, 94 °C 5 sec and 60 °C 30 sec for 40 cycles. The primer sequences of Wnt3a and the endogenous reference GAPDH purchased from Tsingke Biotechnology Co., Ltd. were used in this study as follows: Wnt3a Forward: 5′-CGA GTT TGG GAT GGT GT′; Reverse: 5′-CGA CCA GCA TGT CTT CA-3′. GAPDH Forward: 5′-GGA GTC CAC TGG CGT CTT CA′; Reverse: 5′-GTC ATG AGT CCT TCC ACG ATA CC-3′. mRNA relative quantification was calculated with the Ct(2^−ΔΔCt^) method.

### Immunofluorescence analysis

Cells were seeded on coverslips in 6-well plates, and cultured for 24 h at 37°C. Following fixation with 4% paraformaldehyde (P1110, Solarbio, China) for 20 min, the coverslips were washed three times with PBS for 3 min and gas-permeable membranes with PBS-0.2% Triton X-100 for 10 min. The coverslips were blocked with 5% BSA (SW3015, Solarbio, CHN) for 30 min and incubated overnight at 4°C with anti-β-catenin or anti-E-cadherin antibody diluted 1:100. After three rinses in PBS, the cells were incubated for 1 h at room temperature with Alexa Fluor 594-conjugated secondary antibody at a 1:200 dilution. After sealing with anti-fluorescence quenching sealing tablets (with DAPI) (BL739A, Biosharp, China), the cells were mounted on glass slides and visualized using a Leica inverted fluorescence microscope (DMi8, Leica, Germany).

### Nude mouse tumorigenicity and metastasis assay

Six-week-old female nude mice were injected subcutaneously with 0.1 mL of the SiHa cells suspension (4×10^6^ cells for DYNLT3 overexpression, 1×10^6^ cells for DYNLT3-shRNA) into the back flank. After tumor formation, the tumor size was measured every week in two dimensions, and the volume was measured using the formula: V = width^2^ × length × 0.5 (mm^3^). After 4 to 5 weeks, the mice were killed, and the tumors were dissected and weighed. Cervical cancer cells (2×10^6^) were injected into the tail veins of mice. After 4 to 5 weeks, the mice were killed, and the metastasis sites were checked. The abnormal tissues were quantified by *in vivo* imaging with small animal technology. This study was approved by the Committee for the Ethics of Animal Experiments, Wenzhou Medical University.

### Statistical methods

SPSS 25.0 software was used for statistical analyses. All values are expressed as the mean ± standard deviation. Student’s t test and one-way ANOVA were used to analyze the differences between two groups and multiple groups, respectively. A two-tailed *P* value less than 0.05 was considered statistically significant.

## Data Availability

The original contributions presented in the study are included in the article/**Supplementary Material**. Further inquiries can be directed to the corresponding authors.
