# Corrigendum: DYNLT3 overexpression induces apoptosis and inhibits cell growth and migration *via* inhibition of the Wnt pathway and EMT in cervical cancer

**DOI:** 10.3389/fonc.2024.1545180

**Published:** 2025-01-10

**Authors:** Jianan Zhang, Qi Shen, Lu Xia, Xueqiong Zhu, Xuejie Zhu

**Affiliations:** ^1^ Center of Uterine Cancer Diagnosis and Therapy Research of Zhejiang Province, Department of Obstetrics and Gynecology, The Second Affiliated Hospital of Wenzhou Medical University, Wenzhou, China; ^2^ Department of Obstetrics and Gynecology, The First Affiliated Hospital of Wenzhou Medical University, Wenzhou, China

**Keywords:** DYNLT3, cervical cancer, proliferation, apoptosis, invasion, migration

In the published article, a minor error was identified in **Figure 5C**. Due to carelessness during the preparation of the figures, the panels labeled *“Invasion, SiHa, sh2-Dynlt3”* and *“Migration, CaSki, sh1-Dynlt3”* were incorrectly pasted. The corrected **Figure 5** and its caption appear below.

**Figure 5:**
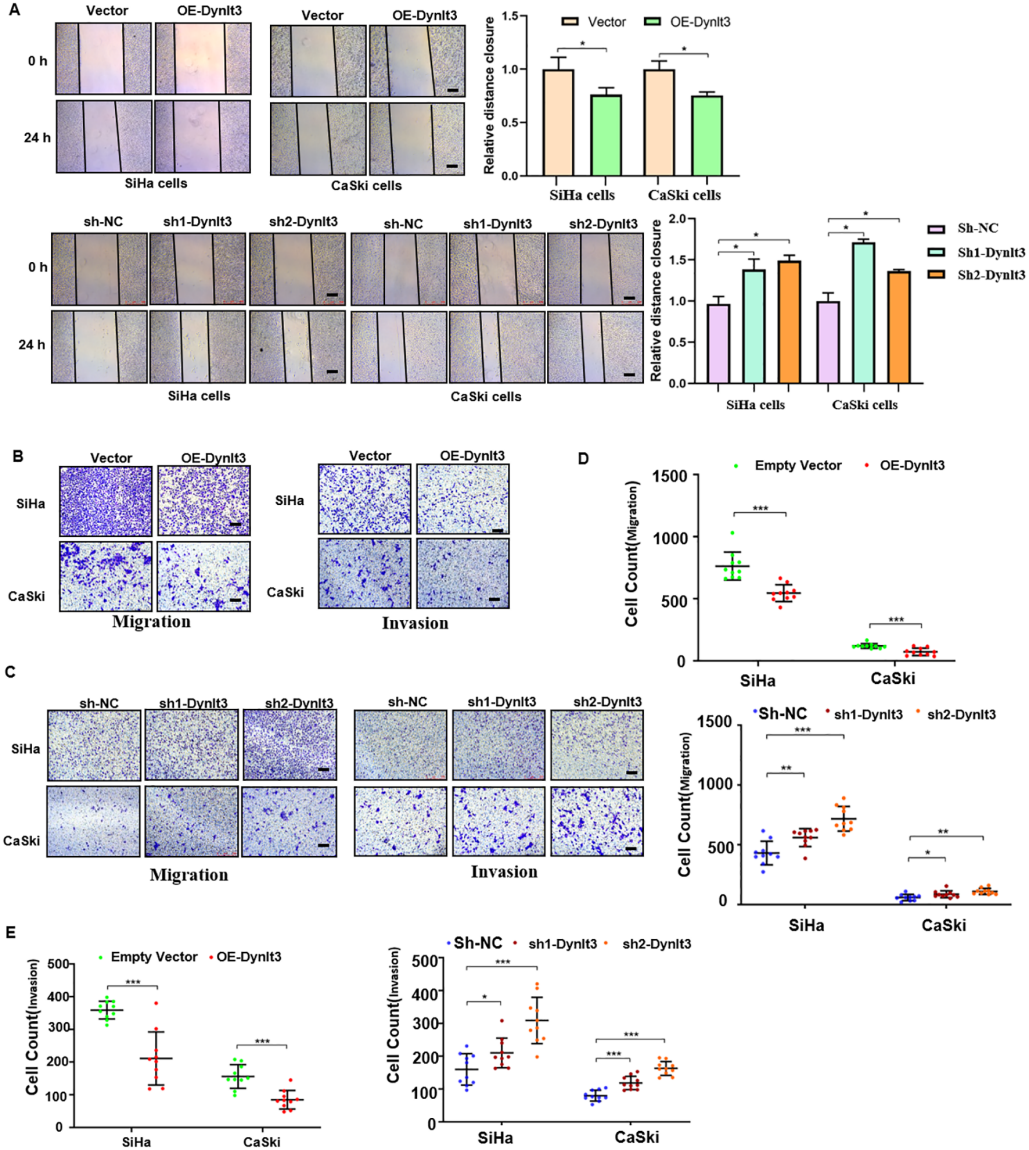
Effects of DYNLT3 on the migration and invasion of cervical cancer cells. **(A)** Left panel: The effects of DYNLT3 on the migration of cervical cancer cells were detected by wound healing assay. Right panel: The quantification of wound closure is shown. **(B)** The effects of DYNLT3 overexpression on the migration and invasion of cervical cancer cells were measured by Transwell assay. **(C)** The effects of DYNLT3 knockdown on the migration and invasion of cervical cancer cells were detected by Transwell assay. Scale bar: 250 μM. **(D)** The quantification of cell migration is illustrated. *P < 0.05, **P < 0.01, ***P < 0.001. **(E)** The quantification of cell invasion is presented. *P < 0.05, **P < 0.01, ***P < 0.001.

The authors apologize for this error and state that this does not change the scientific conclusions of the article in any way. The original article has been updated.

