# Supplementary figures and images for: DYNLT3 overexpression induces apoptosis and inhibits cell growth and migration via inhibition of the Wnt pathway and EMT in cervical cancer
Source: Front Oncol. 2022 Jul 29;12:889238. doi: 10.3389/fonc.2022.889238 (PMC9372440; doi:10.3389/fonc.2022.889238)

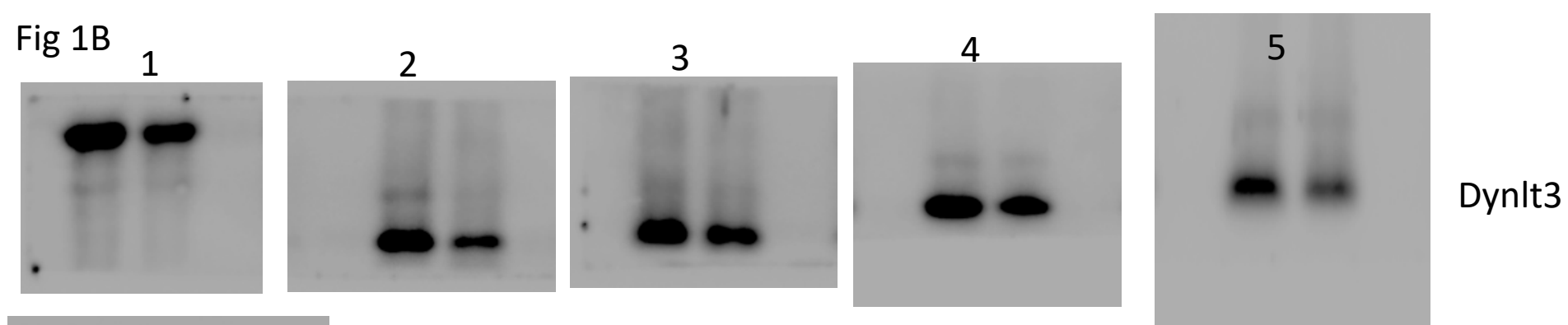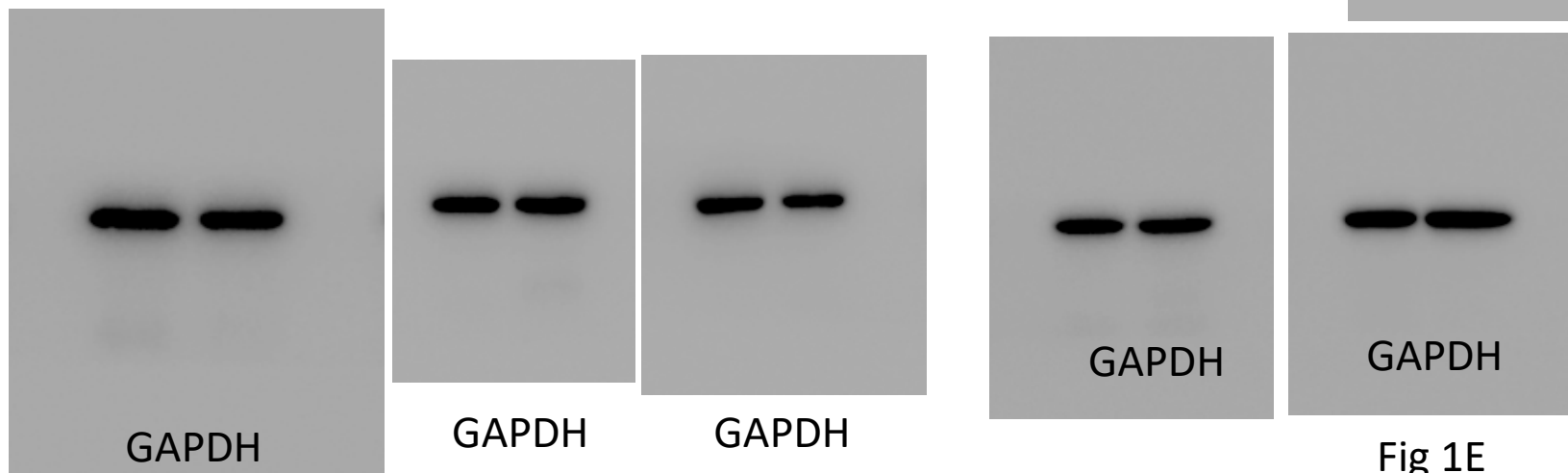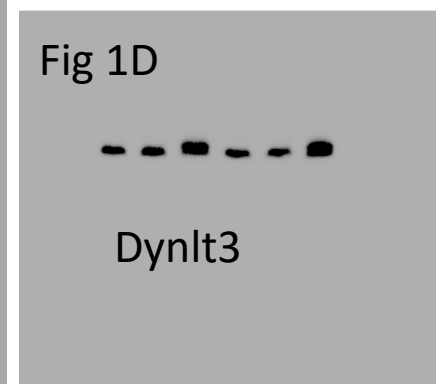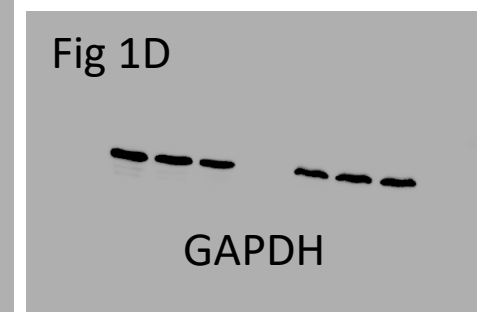

Fig 1E

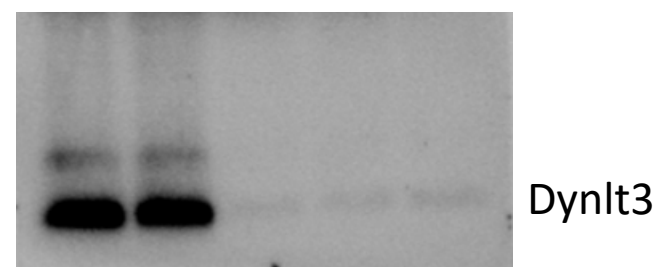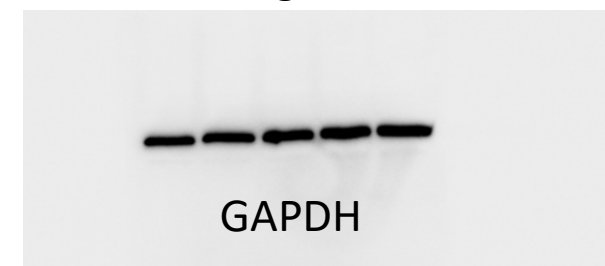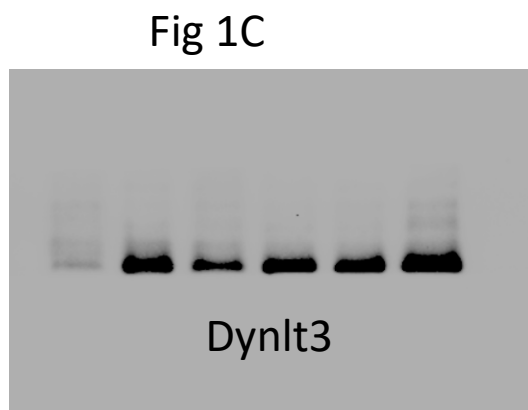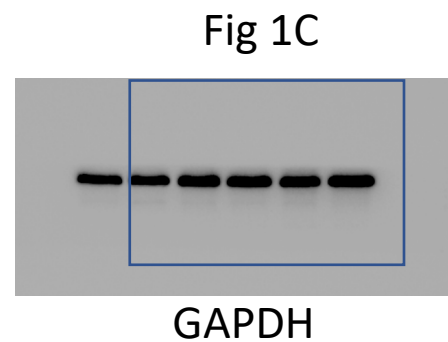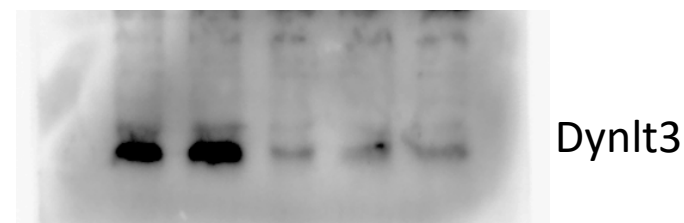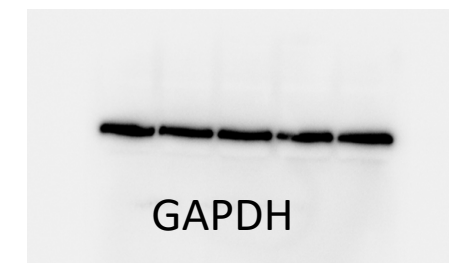

Fig 3E

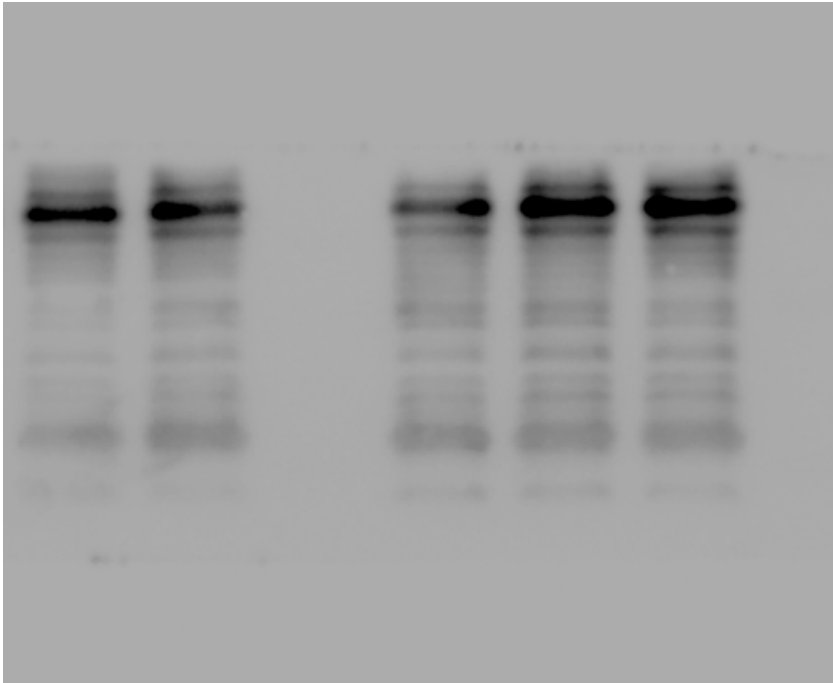

caspase 3

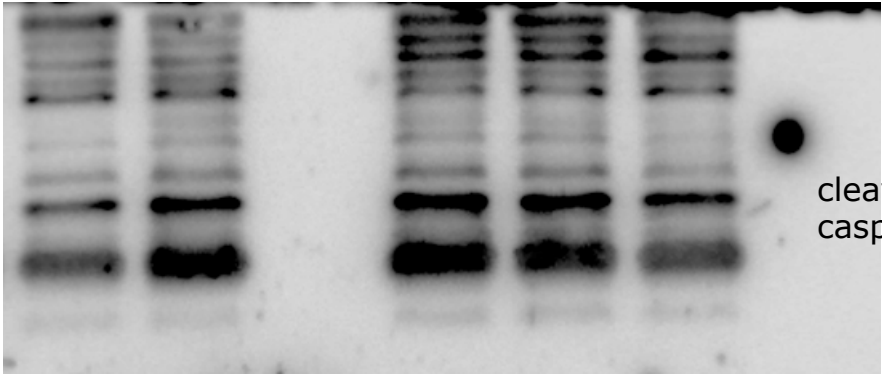

cleaved  
caspase 3

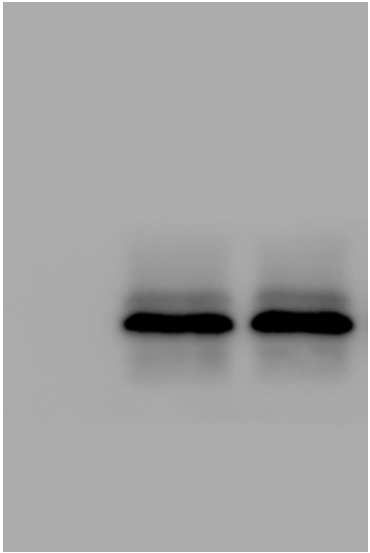

GAPDH

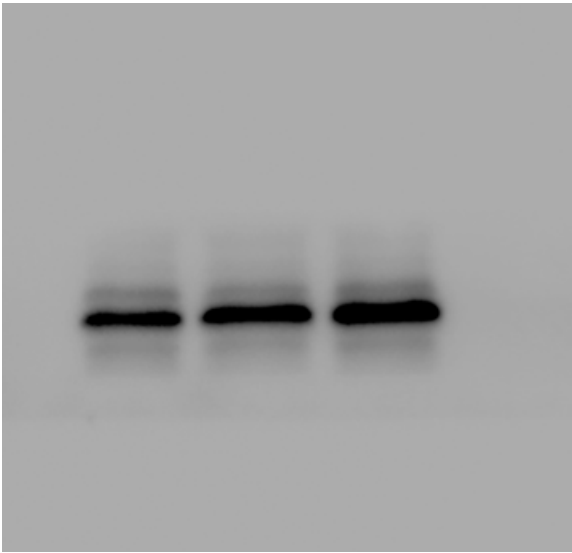

GAPDH

Fig 5A

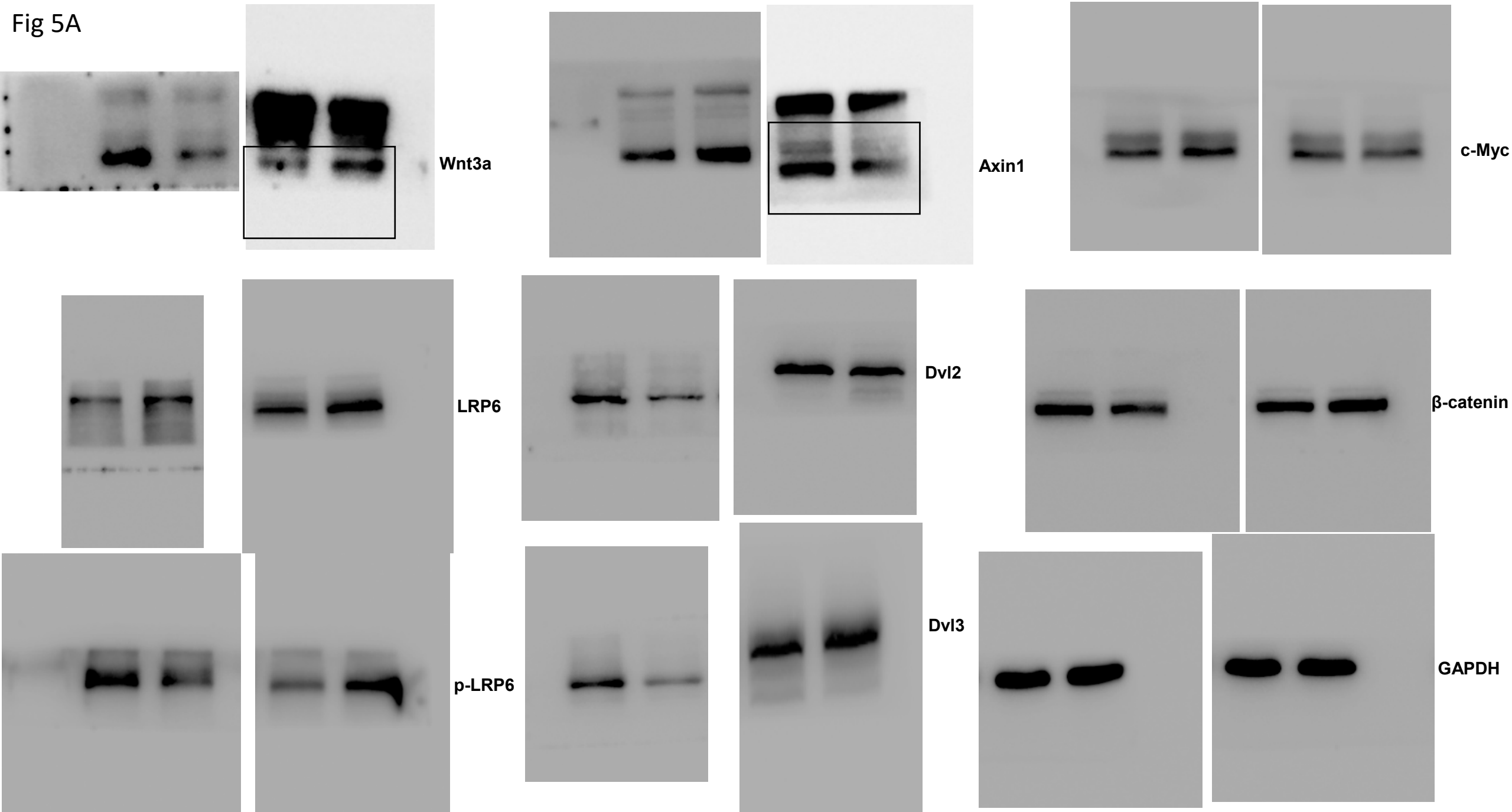

Fig 5B

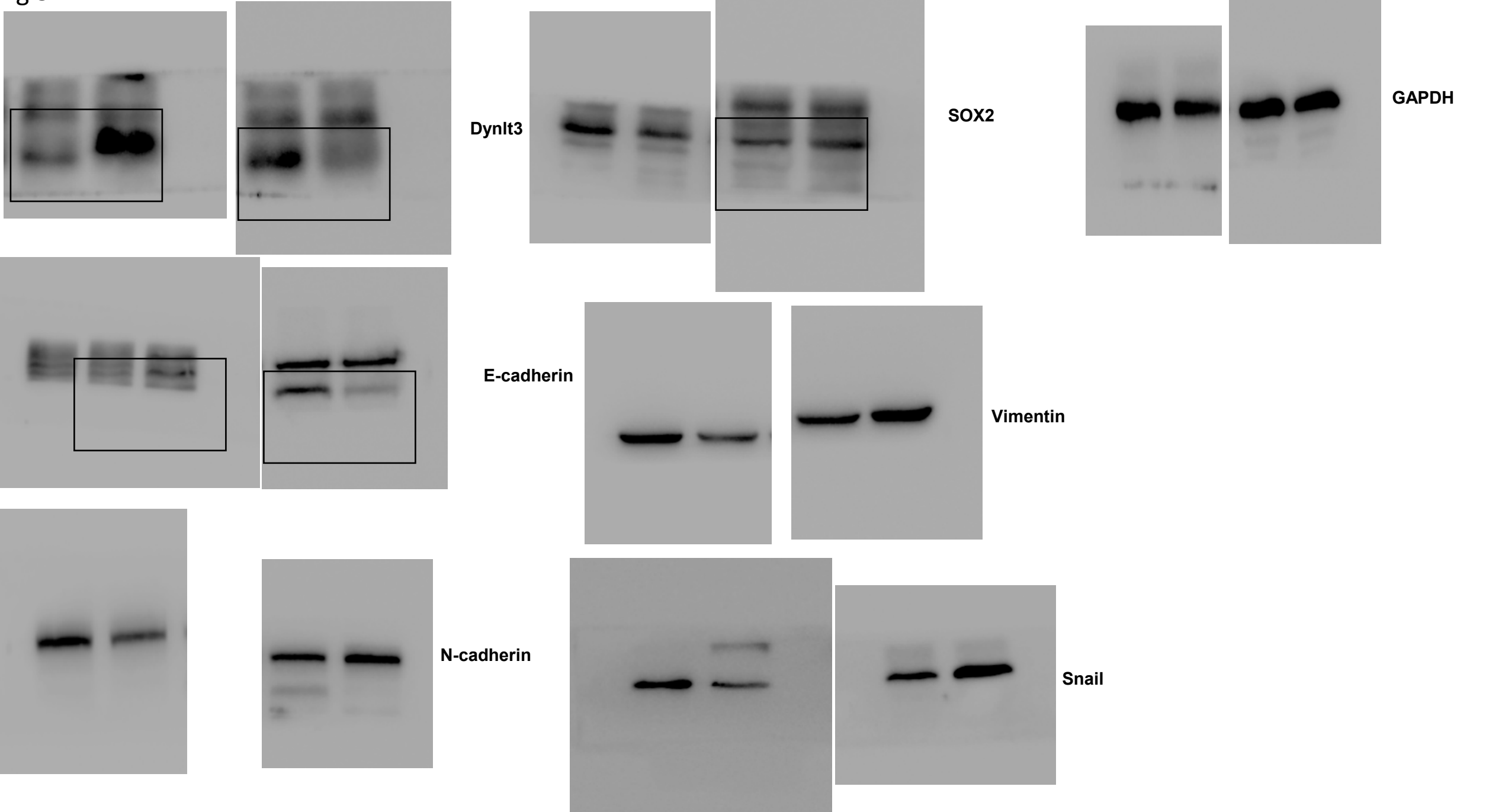

Supplement: Supplementary file 1 [file DataSheet1.pdf]
