# Supplementary material for: DYNLT3 overexpression induces apoptosis and inhibits cell growth and migration via inhibition of the Wnt pathway and EMT in cervical cancer
Source: Front Oncol. 2022 Jul 29;12:889238. doi: 10.3389/fonc.2022.889238 (PMC9372440; doi:10.3389/fonc.2022.889238)
Supplement: Supplementary file 2 [file DataSheet2.pdf]

Figure 1C

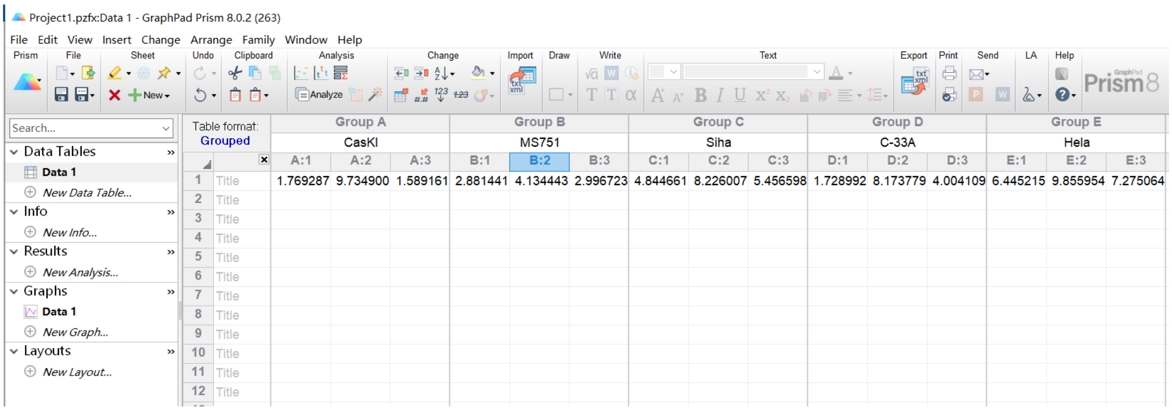

Project1.pzfx:Data 1 - GraphPad Prism 8.0.2 (263)

Table format: Grouped

	Group A			Group B			Group C			Group D			Group E		
	CasKI			MS751			Siha			C-33A			Hela		
	A:1	A:2	A:3	B:1	B:2	B:3	C:1	C:2	C:3	D:1	D:2	D:3	E:1	E:2	E:3
1 Title	1.769287	9.734900	1.589161	2.881441	4.134443	2.996723	4.844661	8.226007	5.456598	1.728992	8.173779	4.004109	6.445215	9.855954	7.275064

Fig 2A

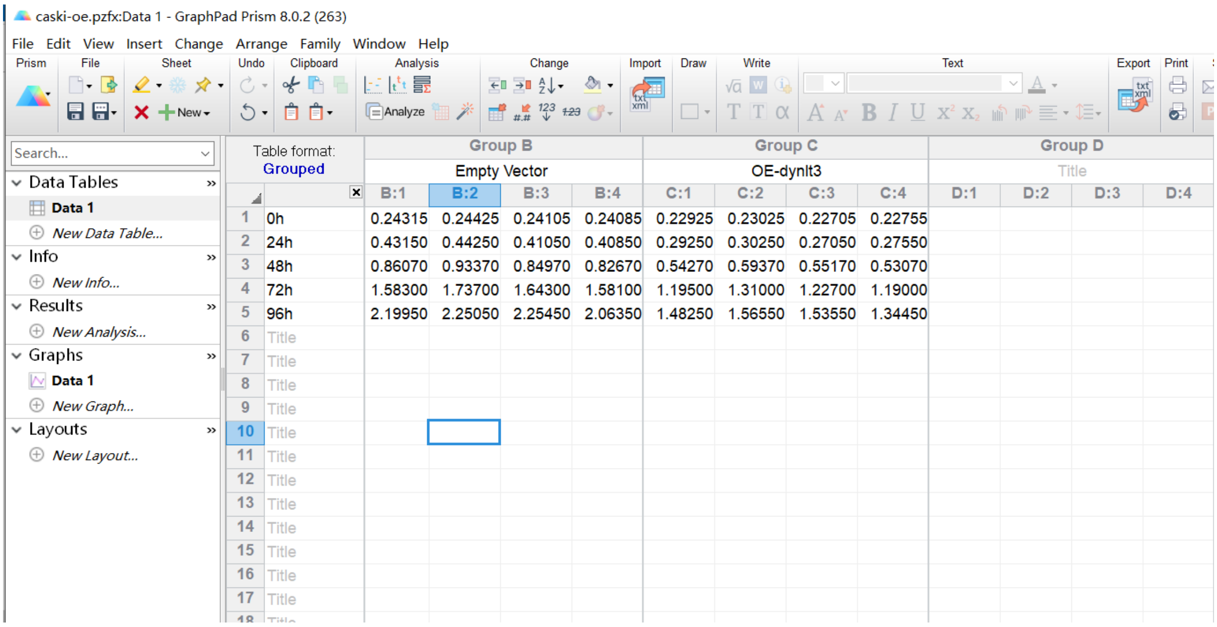

caski-oe.pzfx:Data 1 - GraphPad Prism 8.0.2 (263)

Table format: Grouped

	Group B				Group C				Group D			
	Empty Vector				OE-dynlt3				Title			
	B:1	B:2	B:3	B:4	C:1	C:2	C:3	C:4	D:1	D:2	D:3	D:4
1 0h	0.24315	0.24425	0.24105	0.24085	0.22925	0.23025	0.22705	0.22755				
2 24h	0.43150	0.44250	0.41050	0.40850	0.29250	0.30250	0.27050	0.27550				
3 48h	0.86070	0.93370	0.84970	0.82670	0.54270	0.59370	0.55170	0.53070				
4 72h	1.58300	1.73700	1.64300	1.58100	1.19500	1.31000	1.22700	1.19000				
5 96h	2.19950	2.25050	2.25450	2.06350	1.48250	1.56550	1.53550	1.34450				

caski-sh.pzfx:Data 1 - GraphPad Prism 8.0.2 (263)

Table format: Grouped

	Group A				Group B				Group C			
	Caski-NC				Caski-sh1				Caski-sh2			
	A:1	A:2	A:3	A:4	B:1	B:2	B:3	B:4	C:1	C:2	C:3	C:4
0h	0.162	0.152	0.175	0.200	0.194	0.188	0.197	0.205	0.240	0.231	0.242	0.234
24h	0.236	0.269	0.260	0.287	0.329	0.306	0.343	0.335	0.315	0.353	0.390	0.339
48h	0.495	0.497	0.492	0.516	0.600	0.606	0.607	0.578	0.637	0.642	0.611	0.707
72h	0.990	1.023	1.025	1.097	1.260	1.187	1.193	1.294	1.482	1.373	1.367	1.556
96h	1.262	1.284	1.166	1.222	1.392	1.430	1.325	1.417	1.877	1.609	1.573	1.864

siha-oe.pzfx:Data 1 - GraphPad Prism 8.0.2 (263)

Table format: Grouped

	Group A						Group B						Group C					
	Wild Type						Empty Vector						OE-Dynlt3					
	A:1	A:2	A:3	A:4	A:5	A:6	B:1	B:2	B:3	B:4	B:5	B:6	C:1	C:2	C:3	C:4	C:5	C:6
0h	0.49275	0.50375	0.50875	0.49975	0.49975	0.49286	0.57675	0.51975	0.57175	0.55375	0.57975	0.53275	0.48575	0.46975	0.48575	0.49875	0.45475	0.43475
24h	0.84375	0.84075	0.84675	0.80175	0.81275	0.83569	0.96175	0.87175	0.93675	0.90975	0.91775	0.89975	0.63775	0.60375	0.61375	0.62875	0.55875	0.53075
48h	1.11900	1.14500	1.15500	1.11900	1.03500	1.01200	1.12700	1.11900	1.08900	1.16900	1.14100	1.06400	0.77800	0.79800	0.78800	0.78000	0.75600	0.77600
72h	2.05700	1.94200	2.05000	2.10100	1.95600	2.01600	1.95200	1.95100	1.92400	1.89500	1.91900	1.93100	1.39900	1.43800	1.35200	1.46700	1.40400	1.36400
96h	3.68620	3.68020	3.65620	3.67420	3.70220	3.75920	3.64120	3.59220	3.65120	3.62520	3.55020	3.63820	3.27220	3.47020	3.37020	3.35320	3.33420	3.37920

siha-sh.pzfx:Data 1 - GraphPad Prism 8.0.2 (263)

Table format: Grouped

	Group A				Group B				Group C			
	Siha-NC				Siha-sh1				Siha-sh4			
	A:1	A:2	A:3	A:4	B:1	B:2	B:3	B:4	C:1	C:2	C:3	C:4
0h	0.302	0.336	0.345	0.340	0.370	0.425	0.453	0.445	0.396	0.389	0.409	0.438
24h	0.474	0.498	0.501	0.453	0.514	0.554	0.534	0.576	0.707	0.739	0.648	0.672
48h	0.781	0.839	0.859	0.834	1.017	1.042	1.032	0.995	0.968	0.939	0.924	0.970
72h	1.378	1.499	1.431	1.330	2.046	2.190	2.227	2.108	1.905	2.107	2.004	1.941
96h	2.035	2.250	2.240	2.059	2.767	2.797	3.012	2.873	2.735	2.551	2.747	2.835

Fig 2B

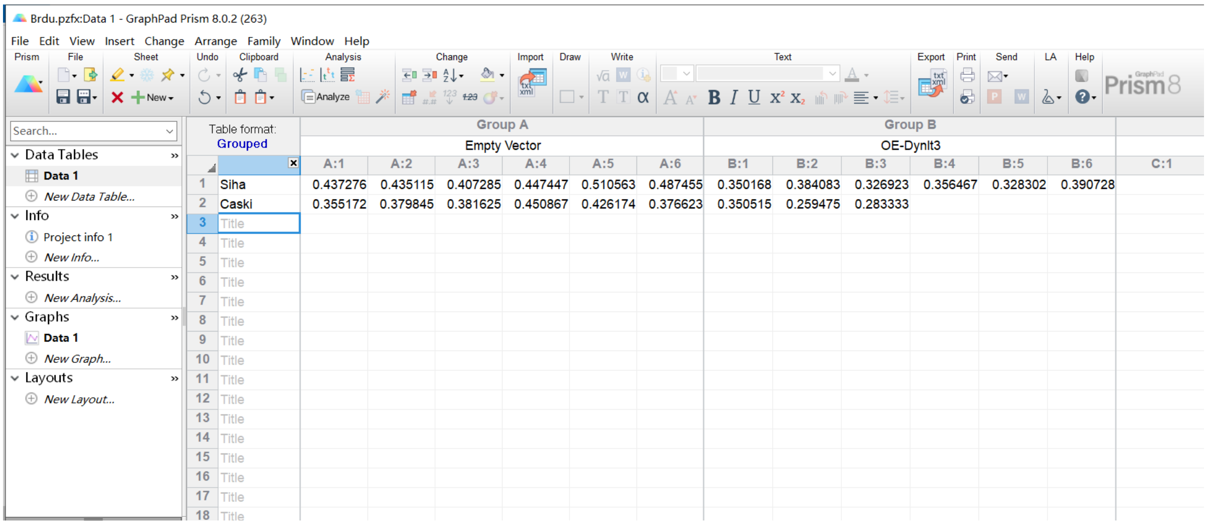

Table format: Grouped	Group A: Empty Vector						Group B: OE-DynIt3					
	A:1	A:2	A:3	A:4	A:5	A:6	B:1	B:2	B:3	B:4	B:5	B:6
Siha	0.437276	0.435115	0.407285	0.447447	0.510563	0.487455	0.350168	0.384083	0.326923	0.356467	0.328302	0.390728
Caski	0.355172	0.379845	0.381625	0.450867	0.426174	0.376623	0.350515	0.259475	0.283333			

Fig 2C

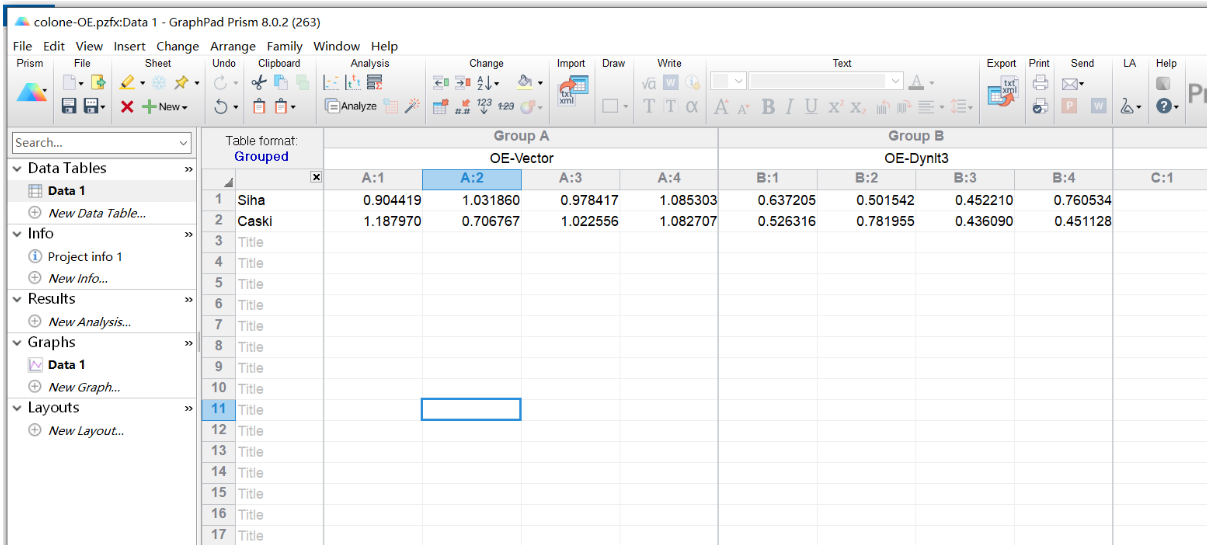

Table format: Grouped	Group A: OE-Vector				Group B: OE-DynIt3			
	A:1	A:2	A:3	A:4	B:1	B:2	B:3	B:4
Siha	0.904419	1.031860	0.978417	1.085303	0.637205	0.501542	0.452210	0.760534
Caski	1.187970	0.706767	1.022556	1.082707	0.526316	0.781955	0.436090	0.451128

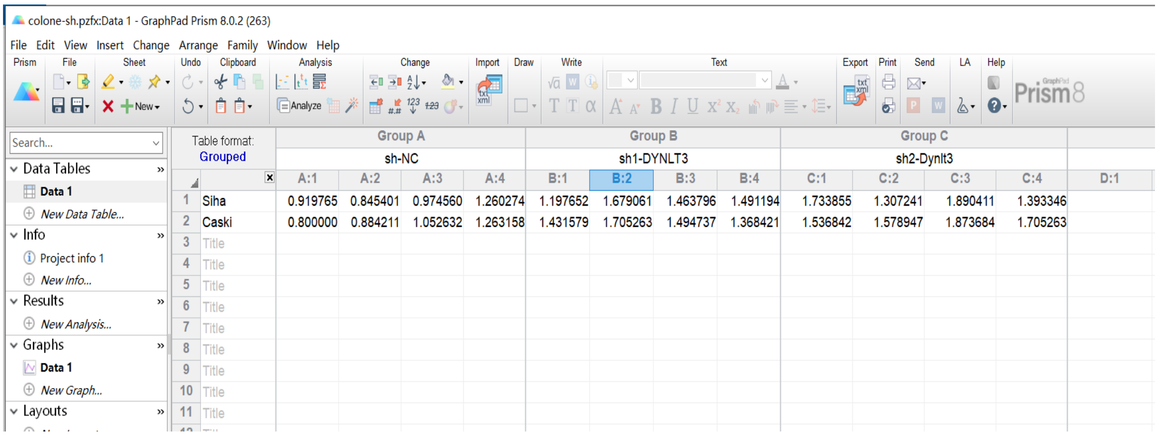

	Group A				Group B				Group C			
	sh-NC				sh1-DYNLT3				sh2-Dynlt3			
	A:1	A:2	A:3	A:4	B:1	B:2	B:3	B:4	C:1	C:2	C:3	C:4
1 Siha	0.919765	0.845401	0.974560	1.260274	1.197652	1.679061	1.463796	1.491194	1.733855	1.307241	1.890411	1.393346
2 Caski	0.800000	0.884211	1.052632	1.263158	1.431579	1.705263	1.494737	1.368421	1.536842	1.578947	1.873684	1.705263

Fig 3C

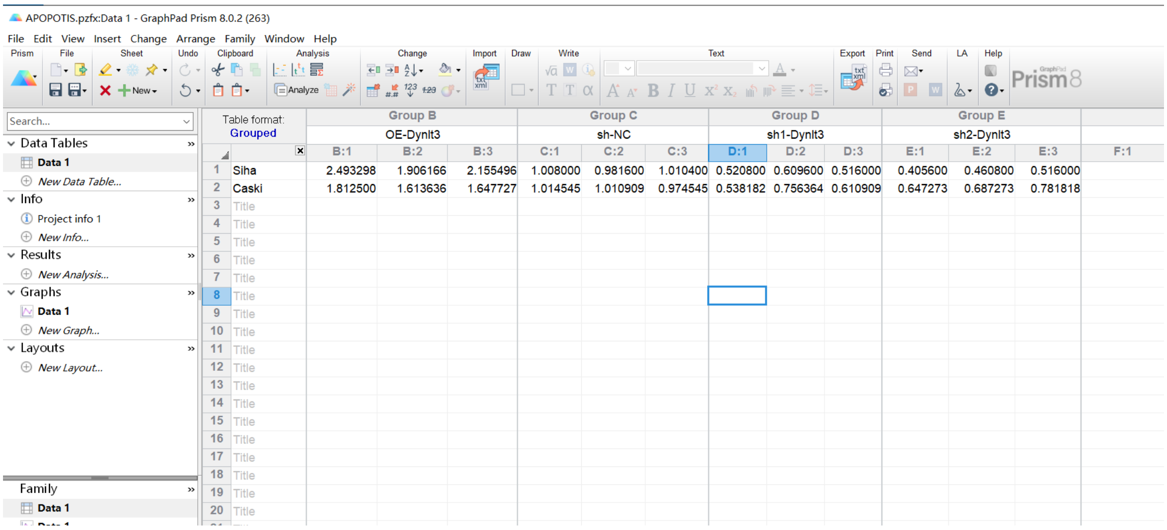

	Group B			Group C			Group D			Group E		
	OE-Dynlt3			sh-NC			sh1-Dynlt3			sh2-Dynlt3		
	B:1	B:2	B:3	C:1	C:2	C:3	D:1	D:2	D:3	E:1	E:2	E:3
1 Siha	2.493298	1.906166	2.155496	1.008000	0.981600	1.010400	0.520800	0.609600	0.516000	0.405600	0.460800	0.516000
2 Caski	1.812500	1.613636	1.647727	1.014545	1.010909	0.974545	0.538182	0.756364	0.610909	0.647273	0.687273	0.781818

Fig 3D

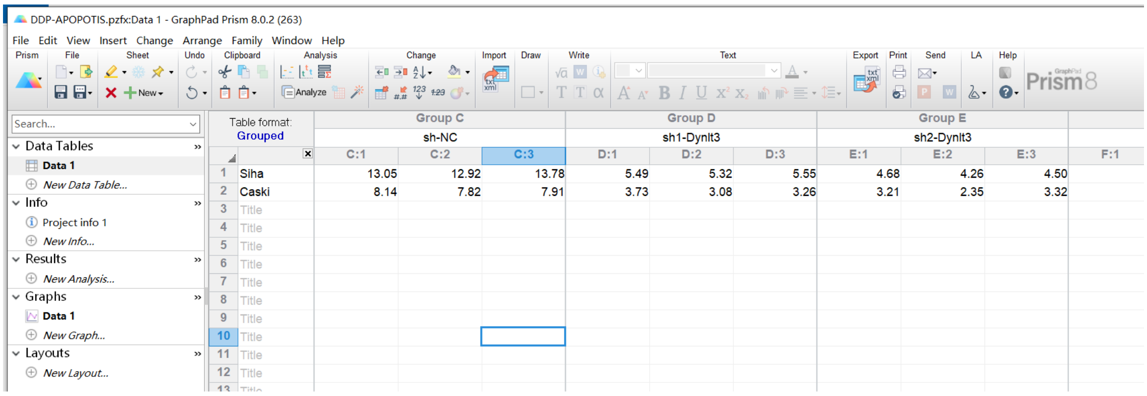

	Group C			Group D			Group E		
	sh-NC			sh1-Dynlt3			sh2-Dynlt3		
	C:1	C:2	C:3	D:1	D:2	D:3	E:1	E:2	E:3
1 Siha	13.05	12.92	13.78	5.49	5.32	5.55	4.68	4.26	4.50
2 Caski	8.14	7.82	7.91	3.73	3.08	3.26	3.21	2.35	3.32

Fig 3E

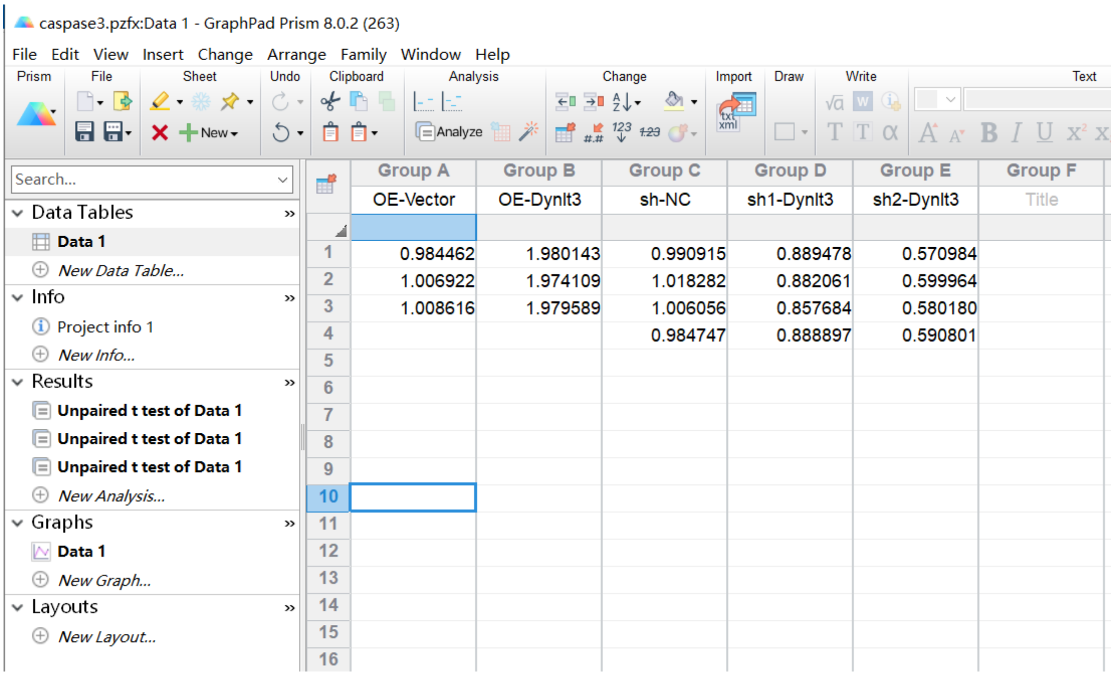

	Group A	Group B	Group C	Group D	Group E	Group F
	OE-Vector	OE-Dynlt3	sh-NC	sh1-Dynlt3	sh2-Dynlt3	Title
1	0.984462	1.980143	0.990915	0.889478	0.570984	
2	1.006922	1.974109	1.018282	0.882061	0.599964	
3	1.008616	1.979589	1.006056	0.857684	0.580180	
4			0.984747	0.888897	0.590801	

Fig 4A

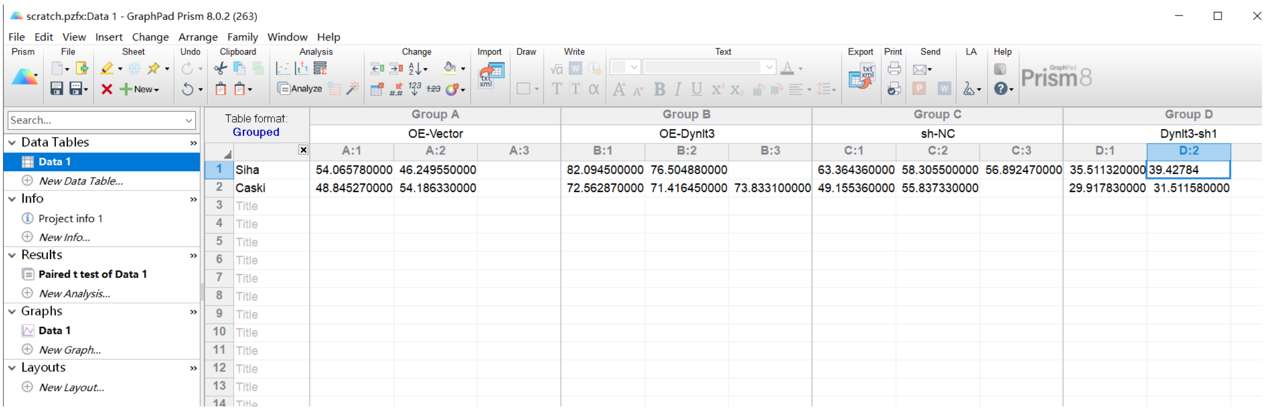

		Group A			Group B			Group C			Group D	
		OE-Vector			OE-Dynlt3			sh-NC			Dynlt3-sh1	
		A:1	A:2	A:3	B:1	B:2	B:3	C:1	C:2	C:3	D:1	D:2
1	Siha	54.065780000	46.249550000		82.094500000	76.504880000		63.364360000	58.305500000	56.892470000	35.511320000	39.42784
2	Caski	48.845270000	54.186330000		72.562870000	71.416450000	73.833100000	49.155360000	55.837330000		29.917830000	31.511580000

Fig 4D

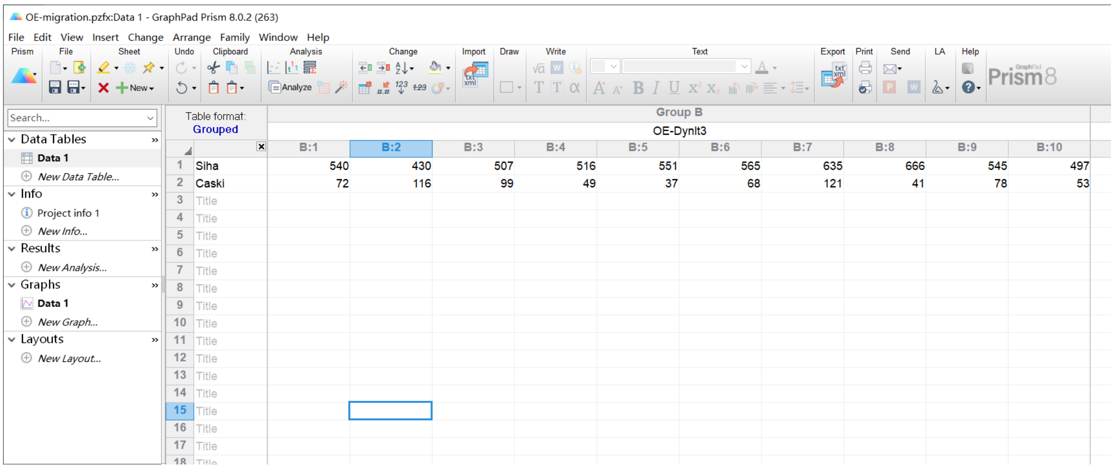

Table format: Grouped	Group B OE-DynIt3									
	B:1	B:2	B:3	B:4	B:5	B:6	B:7	B:8	B:9	B:10
Siha	540	430	507	516	551	565	635	666	545	497
Caski	72	116	99	49	37	68	121	41	78	53

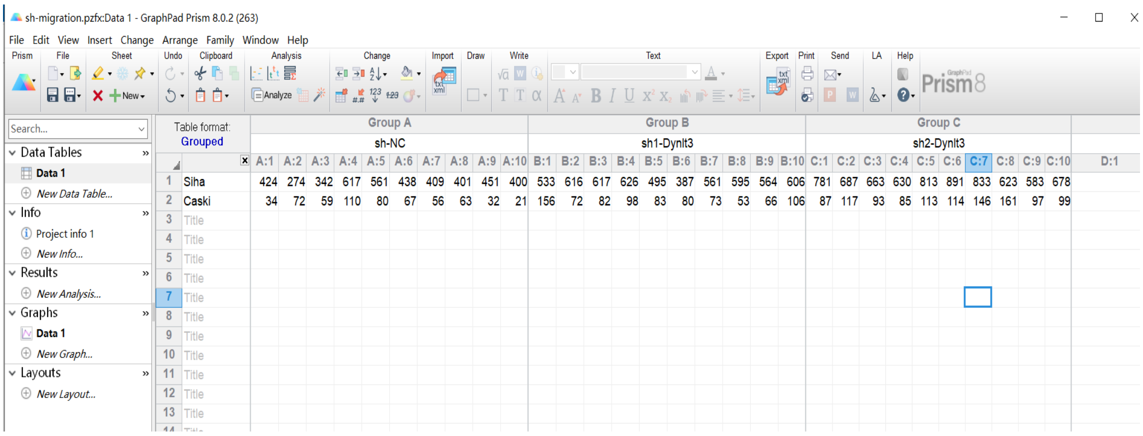

Table format: Grouped	Group A sh-NC									
	A:1	A:2	A:3	A:4	A:5	A:6	A:7	A:8	A:9	A:10
Siha	424	274	342	617	561	438	409	401	451	400
Caski	34	72	59	110	80	67	56	63	32	21

Table format: Grouped	Group B sh1-DynIt3									
	B:1	B:2	B:3	B:4	B:5	B:6	B:7	B:8	B:9	B:10
Siha	533	616	617	626	495	387	561	595	564	606
Caski	156	72	82	98	83	80	73	53	66	106

Table format: Grouped	Group C sh2-DynIt3									
	C:1	C:2	C:3	C:4	C:5	C:6	C:7	C:8	C:9	C:10
Siha	781	687	663	630	813	891	833	623	583	678
Caski	87	117	93	85	113	114	146	161	97	99

Fig 4E

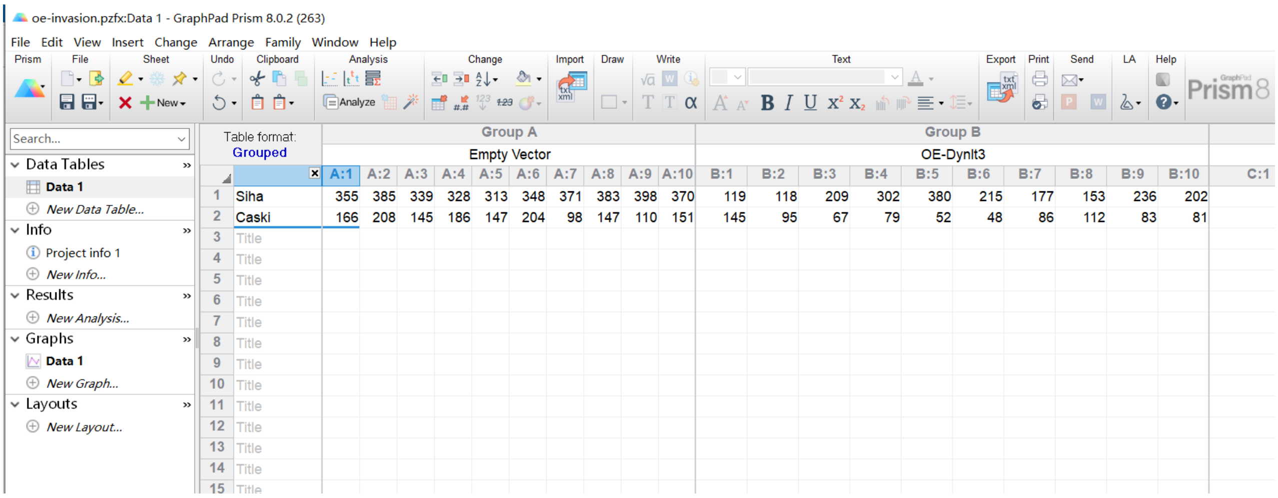

Table format: Grouped	Group A Empty Vector									
	A:1	A:2	A:3	A:4	A:5	A:6	A:7	A:8	A:9	A:10
Siha	355	385	339	328	313	348	371	383	398	370
Caski	166	208	145	186	147	204	98	147	110	151

Table format: Grouped	Group B OE-DynIt3									
	B:1	B:2	B:3	B:4	B:5	B:6	B:7	B:8	B:9	B:10
Siha	119	118	209	302	380	215	177	153	236	202
Caski	145	95	67	79	52	48	86	112	83	81

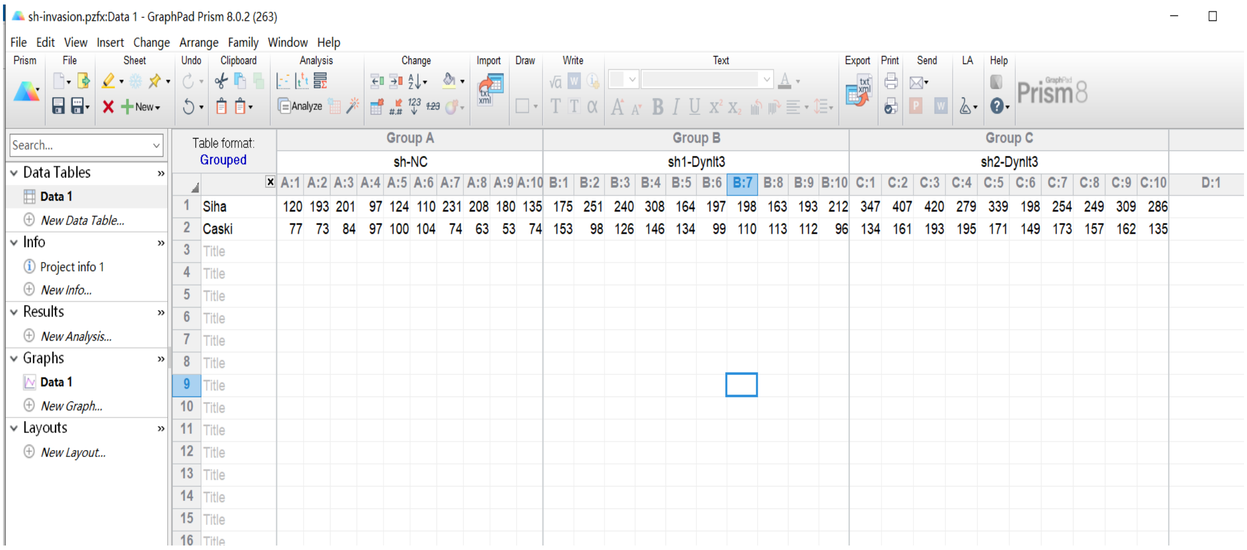

sh-invasion.pzfx:Data 1 - GraphPad Prism 8.0.2 (263)

Table format: Grouped

	Group A									
	sh-NC									
	A:1	A:2	A:3	A:4	A:5	A:6	A:7	A:8	A:9	A:10
Siha	120	193	201	97	124	110	231	208	180	135
Caski	77	73	84	97	100	104	74	63	53	74

	Group B									
	sh1-Dynlt3									
	B:1	B:2	B:3	B:4	B:5	B:6	B:7	B:8	B:9	B:10
Siha	175	251	240	308	164	197	198	163	193	212
Caski	153	98	126	146	134	99	110	113	112	96

	Group C									
	sh2-Dynlt3									
	C:1	C:2	C:3	C:4	C:5	C:6	C:7	C:8	C:9	C:10
Siha	347	407	420	279	339	198	254	249	309	286
Caski	134	161	193	195	171	149	173	157	162	135

Fig 5A

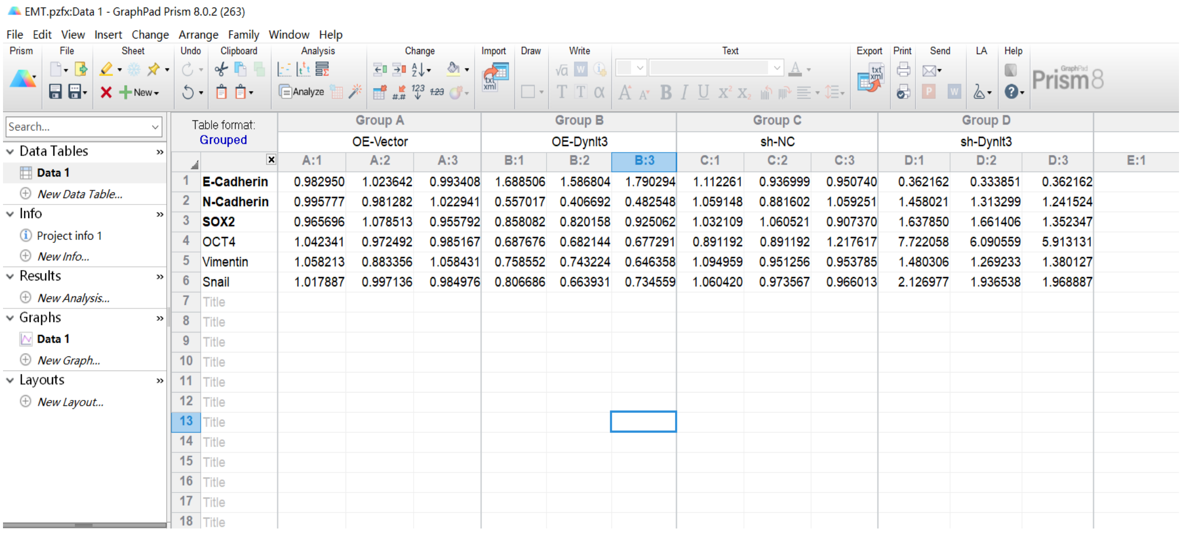

EMT.pzfx:Data 1 - GraphPad Prism 8.0.2 (263)

Table format: Grouped

	Group A			Group B			Group C			Group D		
	OE-Vector			OE-Dynlt3			sh-NC			sh-Dynlt3		
	A:1	A:2	A:3	B:1	B:2	B:3	C:1	C:2	C:3	D:1	D:2	D:3
**E-Cadherin**	0.982950	1.023642	0.993408	1.688506	1.586804	1.790294	1.112261	0.936999	0.950740	0.362162	0.333851	0.362162
**N-Cadherin**	0.995777	0.981282	1.022941	0.557017	0.406692	0.482548	1.059148	0.881602	1.059251	1.458021	1.313299	1.241524
**SOX2**	0.965696	1.078513	0.955792	0.858082	0.820158	0.925062	1.032109	1.060521	0.907370	1.637850	1.661406	1.352347
OCT4	1.042341	0.972492	0.985167	0.687676	0.682144	0.677291	0.891192	0.891192	1.217617	7.722058	6.090559	5.913131
Vimentin	1.058213	0.883356	1.058431	0.758552	0.743224	0.646358	1.094959	0.951256	0.953785	1.480306	1.269233	1.380127
Snail	1.017887	0.997136	0.984976	0.806686	0.663931	0.734559	1.060420	0.973567	0.966013	2.126977	1.936538	1.968887

Fig 5B

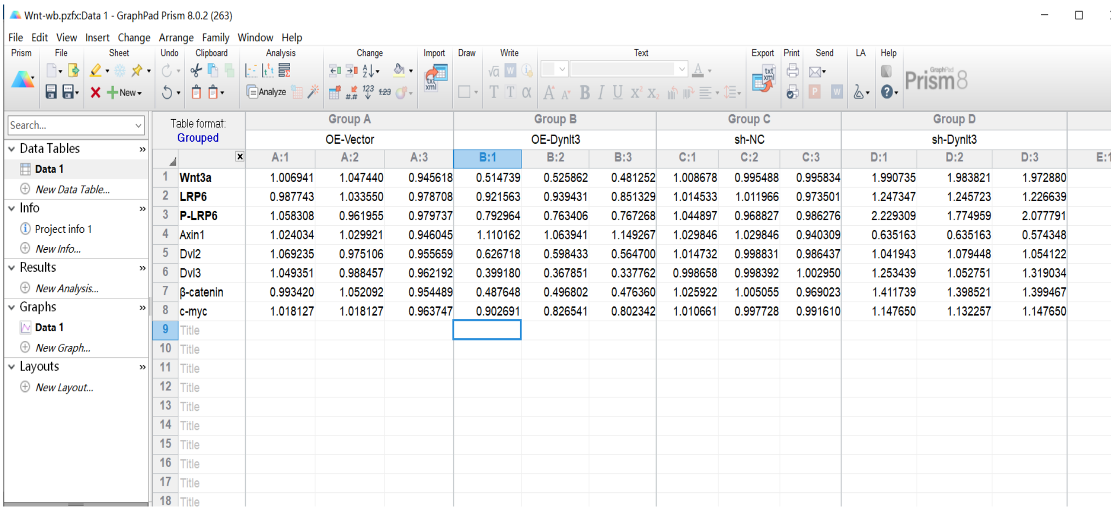

Table format: Grouped	Group A OE-Vector			Group B OE-Dynlt3			Group C sh-NC			Group D sh-Dynlt3		
	A:1	A:2	A:3	B:1	B:2	B:3	C:1	C:2	C:3	D:1	D:2	D:3
1 Wnt3a	1.006941	1.047440	0.945618	0.514739	0.525862	0.481252	1.008678	0.995488	0.995834	1.990735	1.983821	1.972880
2 LRP6	0.987743	1.033550	0.978708	0.921563	0.939431	0.851329	1.014533	1.011966	0.973501	1.247347	1.245723	1.226639
3 P-LRP6	1.058308	0.961955	0.979737	0.792964	0.763406	0.767268	1.044897	0.968827	0.986276	2.229309	1.774959	2.077791
4 Axin1	1.024034	1.029921	0.946045	1.110162	1.063941	1.149267	1.029846	1.029846	0.940309	0.635163	0.635163	0.574348
5 Dvl2	1.069235	0.975106	0.955659	0.626718	0.598433	0.564700	1.014732	0.998831	0.986437	1.041943	1.079448	1.054122
6 Dvl3	1.049351	0.988457	0.962192	0.399180	0.367851	0.337762	0.998658	0.998392	1.002950	1.253439	1.052751	1.319034
7 β-catenin	0.993420	1.052092	0.954489	0.487648	0.496802	0.476360	1.025922	1.005055	0.969023	1.411739	1.398521	1.399467
8 c-myc	1.018127	1.018127	0.963747	0.902691	0.826541	0.802342	1.010661	0.997728	0.991610	1.147650	1.132257	1.147650

Fig 5C

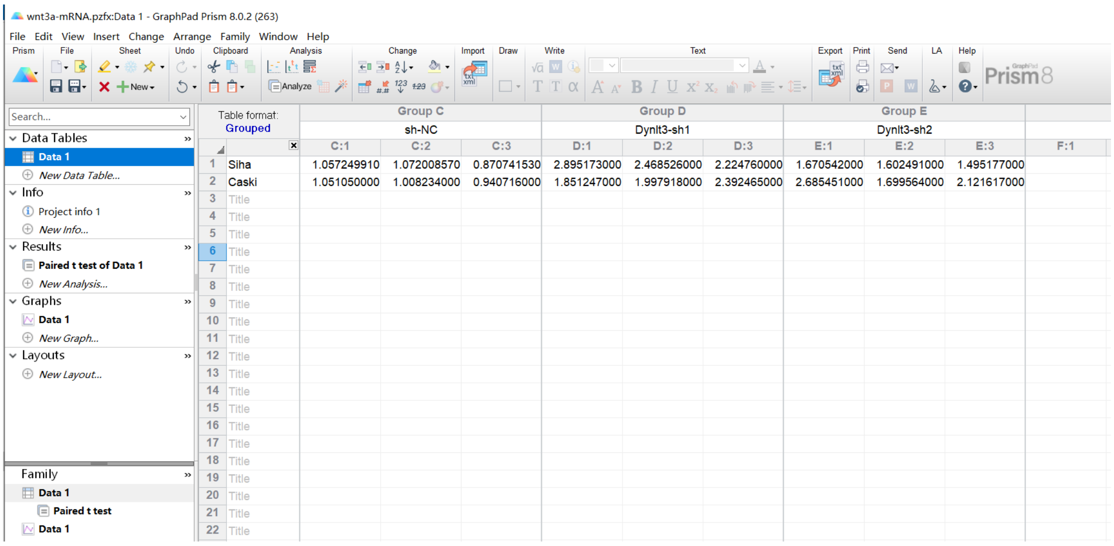

Table format: Grouped	Group C sh-NC			Group D Dynlt3-sh1			Group E Dynlt3-sh2		
	C:1	C:2	C:3	D:1	D:2	D:3	E:1	E:2	E:3
1 Siha	1.057249910	1.072008570	0.870741530	2.895173000	2.468526000	2.224760000	1.670542000	1.602491000	1.495177000
2 Caski	1.051050000	1.008234000	0.940716000	1.851247000	1.997918000	2.392465000	2.685451000	1.699564000	2.121617000

Fig 7A

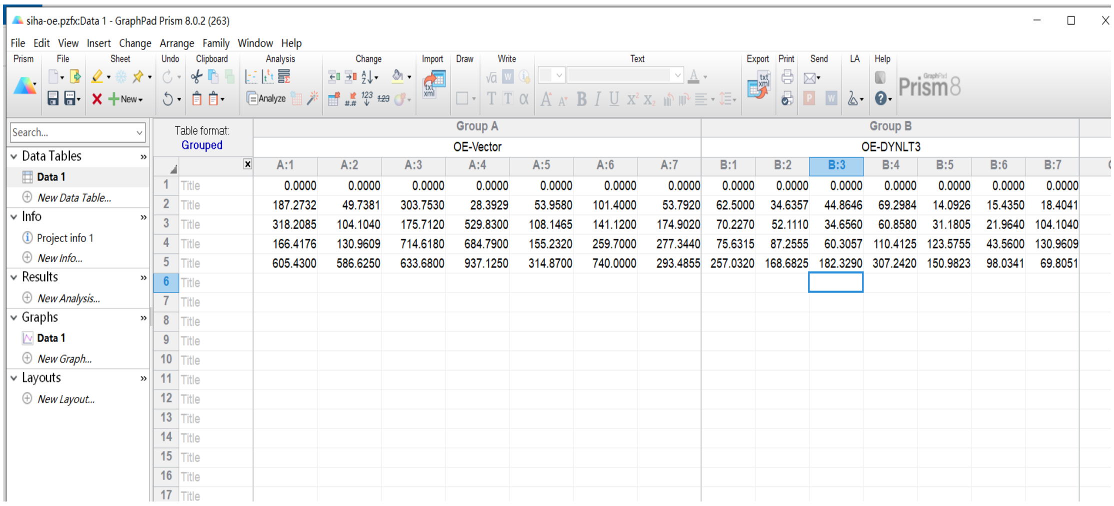

	Group A OE-Vector							Group B OE-DYNLT3						
	A:1	A:2	A:3	A:4	A:5	A:6	A:7	B:1	B:2	B:3	B:4	B:5	B:6	B:7
1 Title	0.0000	0.0000	0.0000	0.0000	0.0000	0.0000	0.0000	0.0000	0.0000	0.0000	0.0000	0.0000	0.0000	0.0000
2 Title	187.2732	49.7381	303.7530	28.3929	53.9580	101.4000	53.7920	62.5000	34.6357	44.8646	69.2984	14.0926	15.4350	18.4041
3 Title	318.2085	104.1040	175.7120	529.8300	108.1465	141.1200	174.9020	70.2270	52.1110	34.6560	60.8580	31.1805	21.9640	104.1040
4 Title	166.4176	130.9609	714.6180	684.7900	155.2320	259.7000	277.3440	75.6315	87.2555	60.3057	110.4125	123.5755	43.5600	130.9609
5 Title	605.4300	586.6250	633.6800	937.1250	314.8700	740.0000	293.4855	257.0320	168.6825	182.3290	307.2420	150.9823	98.0341	69.8051

Fig 7B

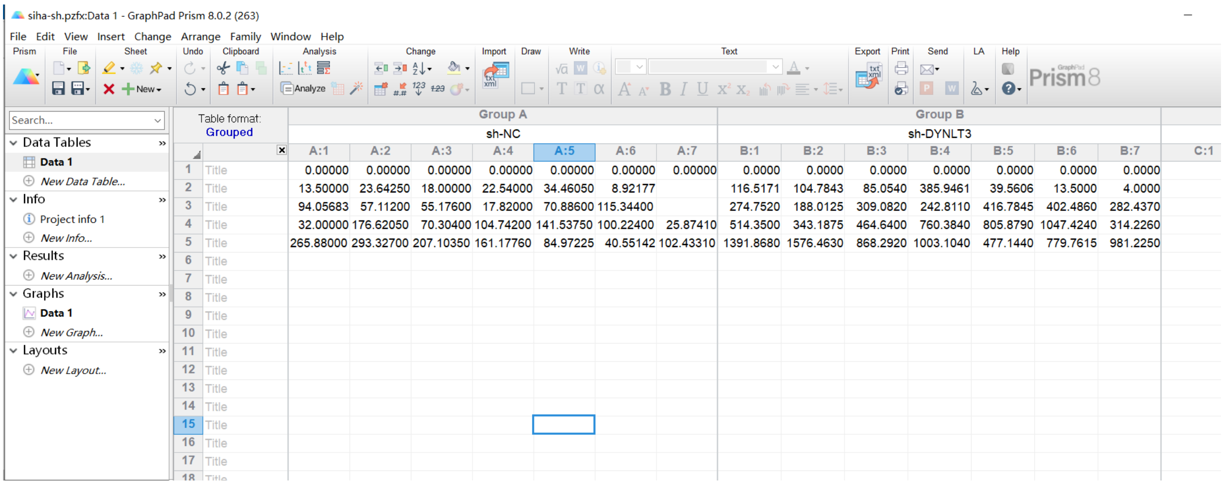

	Group A sh-NC							Group B sh-DYNLT3						
	A:1	A:2	A:3	A:4	A:5	A:6	A:7	B:1	B:2	B:3	B:4	B:5	B:6	B:7
1 Title	0.00000	0.00000	0.00000	0.00000	0.00000	0.00000	0.00000	0.0000	0.0000	0.0000	0.0000	0.0000	0.0000	0.0000
2 Title	13.50000	23.64250	18.00000	22.54000	34.46050	8.92177		116.5171	104.7843	85.0540	385.9461	39.5606	13.5000	4.0000
3 Title	94.05683	57.11200	55.17600	17.82000	70.88600	115.34400		274.7520	188.0125	309.0820	242.8110	416.7845	402.4860	282.4370
4 Title	32.00000	176.62050	70.30400	104.74200	141.53750	100.22400	25.87410	514.3500	343.1875	464.6400	760.3840	805.8790	1047.4240	314.2260
5 Title	265.88000	293.32700	207.10350	161.17760	84.97225	40.55142	102.43310	1391.8680	1576.4630	868.2920	1003.1040	477.1440	779.7615	981.2250
